# The developmental and evolutionary characteristics of transcription factor binding site clustered regions based on an explainable machine learning model

**DOI:** 10.1093/nar/gkae441

**Published:** 2024-05-30

**Authors:** Zhangyi Ouyang, Feng Liu, Wanying Li, Junting Wang, Bijia Chen, Yang Zheng, Yaru Li, Huan Tao, Xiang Xu, Cheng Li, Yuwen Cong, Hao Li, Xiaochen Bo, Hebing Chen

**Affiliations:** Academy of Military Medical Sciences, Beijing 100850, China; College of Medical Informatics, Chongqing Medical University, Chongqing 400016, China; Academy of Military Medical Sciences, Beijing 100850, China; Academy of Military Medical Sciences, Beijing 100850, China; Academy of Military Medical Sciences, Beijing 100850, China; Academy of Military Medical Sciences, Beijing 100850, China; Academy of Military Medical Sciences, Beijing 100850, China; Academy of Military Medical Sciences, Beijing 100850, China; Academy of Military Medical Sciences, Beijing 100850, China; Center for Bioinformatics, School of Life Sciences, Center for Statistical Science, Peking University, Beijing 100871, China; Academy of Military Medical Sciences, Beijing 100850, China; Academy of Military Medical Sciences, Beijing 100850, China; Academy of Military Medical Sciences, Beijing 100850, China; Academy of Military Medical Sciences, Beijing 100850, China

## Abstract

Gene expression is temporally and spatially regulated by the interaction of transcription factors (TFs) and *cis-*regulatory elements (CREs). The uneven distribution of TF binding sites across the genome poses challenges in understanding how this distribution evolves to regulate spatio-temporal gene expression and consequent heritable phenotypic variation. In this study, chromatin accessibility profiles and gene expression profiles were collected from several species including mammals (human, mouse, bovine), fish (zebrafish and medaka), and chicken. Transcription factor binding sites clustered regions (TFCRs) at different embryonic stages were characterized to investigate regulatory evolution. The study revealed dynamic changes in TFCR distribution during embryonic development and species evolution. The synchronization between TFCR complexity and gene expression was assessed across species using RegulatoryScore. Additionally, an explainable machine learning model highlighted the importance of the distance between TFCR and promoter in the coordinated regulation of TFCRs on gene expression. Our results revealed the developmental and evolutionary dynamics of TFCRs during embryonic development from fish, chicken to mammals. These data provide valuable resources for exploring the relationship between transcriptional regulation and phenotypic differences during embryonic development.

## Graphical Abstract

**Graphical Abstract:**
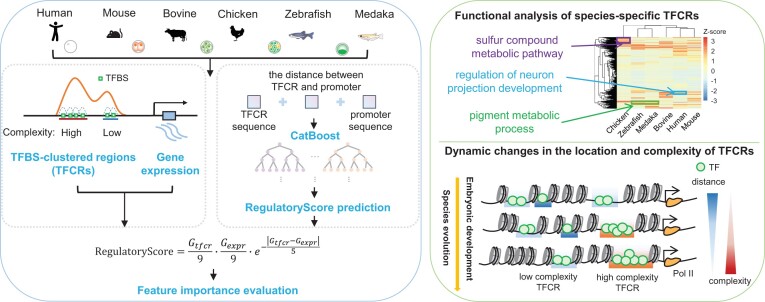

## Introduction

Transcriptional regulation can manage traits by changing temporal and spatial gene expression through the interaction of transcription factors (TFs) and *cis-*regulatory elements (CREs). It plays an important role in organism evolution and phenotypic variation. Some phenotypic differences among species cannot be explained by sequences of protein-coding genes but may result from transcriptional regulation of non-coding regions (1–3). In the evolutionary landscapes of candidate CREs and transcription factor binding sites (TFBSs) across placental mammals, a subset of elements associated with complex traits underwent purifying selection in the mammalian lineage (4).

Protein coding sequence diverges slightly between species with close evolutionary relationships, such as 99% similarity between human and chimpanzee (5). High similarity of protein coding sequence keeps the structural binding domain and function of TF proteins similar. When the binding ability and preference of TF protein remain unchanged, it is still unclear how the non-coding sequence changes to regulate gene expression during evolution.

Transcriptional regulation is a key factor in understanding phenotypic evolution. Embryonic development is also crucial for understanding the phenotypic evolution because morphology changes during development (6). During embryonic development, the binding motifs of TF protein do not change, but the change of CREs contributes to regulating the developmental plasticity. The accumulation of chromatin accessibility data and gene expression profiles during embryonic development has enabled the comparison of transcriptional regulation in various species. Previous studies have reported widespread, accessible chromatin regions in early embryos of human (7), mouse (8), bovine (9) and zebrafish (10). Dynamic chromatin accessibility underlies the transcription-dependent transition during embryonic development (7). While conservation and divergence of the chromatin and epigenomic landscape between species has been well explored (9,11,12), it is still worth investigating how CREs evolve to regulate the spatiotemporal expression of genes between species during embryonic development.

Previous studies observed that TFBSs are highly clustered in the genomes of eukaryotes such as human (13,14), fruit flies (15), and nematodes (16), which may reflect the process of synergistic regulation of gene expression. Several computational methods were developed to identify these clusters of CREs (COREs) from chromatin accessibility profiles (17,18). Our previous work reported the comprehensive map of TFBS-clustered regions (TFCRs) in 133 human cell types using Gaussian kernel density estimation, revealing cell type specificities and lineage programming of TFCRs (17). In addition, Tonekaboni *et al.* showed the genomic structure of these COREs and the enhanced values of COREs over super-enhancers (SEs), suggesting the utility of COREs to discriminate cancer types and underpin biological pathways (18). However, these studies have mainly focused on human transcription factors, somewhat ignoring the differences in the distribution of TFCRs in different species.

In this study, we collected chromatin accessibility profiles and gene expression profiles from mammals (human (7), mouse (8,19), bovine (9)), fish (zebrafish (10,20) and medaka (19)), and chicken (19), and identified the dynamic changes of TFCRs during embryonic development. By cross-species comparison, we investigated the distribution pattern of TFCRs during embryonic development among species. During embryonic development, distance between TFCRs and promoters became closer. During evolution, regulation relationship between TFCRs and genes became more complicated from simple to complex species. Importantly, we evaluated the synchronization of TFCR complexity and gene expression by defining the RegulatoryScore index among species. Genes with high RegulatoryScore in human embryonic stem cells were enriched in neuronal development and synaptic transmission processes, indicating the specific neuron regulatory patterns during human development compared to other species. To find factors affecting RegulatoryScores prediction, the explainable machine learning models were developed. Among all species and developmental stages analyzed, the results showed that distance between TFCRs and gene promoters was the most contributing factor to the regulation of TFCR on gene expression.

## Materials and methods

### Dataset collection

The reference genomes of each species used in this study were downloaded from Ensemble (21): hg19, mm10, bosTau9, galGal5, danRer10, oryLat2, sacCer3, ce10, dmel-r6.12, xenTro9, susScr11.1. ATAC-seq peaks and gene expression data were downloaded from the GEO database (22). The human data were obtained from GSE101571 (7). The mouse data were obtained from GSE66581 (8), GSE66582 (8) and DRP004498 (19). The bovine data were obtained from GSE143658 (9) and GSE52415 (23). The chicken and medaka data were obtained from DRP004498 (19), in which the whole embryo was used to perform ATAC-seq. The amniotic membranes were removed from the staged embryos, and at least two embryos were pooled to prepare each biological replicate. Embryos were minced by using a razor blade, placed in homogenization buffer (25 mM d-sucrose, 20 mM tricine [pH 7.8], 15 mM NaCl, 60 mM KCl, 2 mM MgCl_2_, 0.5 mM spermidine and cOmplete Protease Inhibitor Cocktail Tablet [Roche]), and homogenized in an ice-cold Dounce tissue grinder with a loose-fitting pestle. The data of zebrafish was obtained from GSE101779 (10), GSE106428 (20), GSM1289382 (24), and GSE106431 (20). The data of yeast, *C. elegans*, *D. melanogaster*, frog, pig data was obtained from GSE106450 (25), GSE89608 (26), GSE113240 (27), GSE145619 (28), GSE143288 (29), respectively. The data of primate species was obtained from GSE130871 (30). The reference genomes used for marmoset, macaque, chimpanzee, and human were calJac3, rheMac8, panTro5 and hg38, respectively. The ChIP-seq data for rabbit, cat, dog, opossum, and rat was obtained from E-MTAB-2633 (31), with the reference genomes for these mammals being oryCun2, felCat5, canFam3, monDom5 and rn5. All peaks are identified by MACS2 (32). The sequence depth of ATAC-seq data and the parameters used to call peaks across species are shown in Supplementary Table S1. The effect of peak calling parameters on the distribution of ATAC-seq peaks was evaluated and the distribution of peaks were comparable across species (see Supplementary methods, Supplementary Figure S1, Supplementary Table S2).

Homologous genes were downloaded from Ensemble BioMart (http://www.ensembl.org/biomart/martview). For example, to get human-mouse homologous genes, we choose ‘Homo sapiens genes (NCBI36)’, click on ‘Filters’ in the left menu, unfold the ‘MULTI SPECIES COMPARISONS’ box and tick the ‘Homolog filters’ option, then choose ‘Orthologous Mouse Genes’ from the drop-down menu and click on ‘Homologs’ attributes.

### Identification of TFCRs

First, the TFBSs were identified by FIMO (33) using position-specific weight matrices (PWMs) of transcription factors from CIS-BP databases (34)(Supplementary Table S3). We chose CIS-BP because it included TF motifs across many eukaryotic genomes. Species-specific motifs were used to consider the motif divergence of TFs between species. Genomic sequences under the open chromatin regions were used as inputs for FIMO with a custom library of all motifs for each species to scan for motif instances at a *P*-value threshold of 10^−5^. Average numbers of identified TFBSs located within 5 kb genomic bins were significantly higher compared to shuffled TFBSs among all species (Wilcoxon rank-sum test *P*-value < 0.0001), especially in human (Supplementary Table S4). This result showed that identified TFBSs were more closely distributed than randomly shuffled TFBSs (Supplementary Figure S2), indicating the clustering characteristic of TFBSs in those species. Then, an established method (17,35) was used to identify TFCRs by performing the Gaussian kernel density estimations across the genome (with a bandwidth of 300 bp centered on each TFBS). Each peak in density profile was considered a TFCR. To determine the complexity of each TFCR, the Gaussian kernelized distances from each peak that contributed at least 0.1 to its strength were determined. The complexity of each TFCR was determined by the quantity and proximity of the contributing TFBS (see Supplementary methods). Robustness of TFCRs identification was evaluated under different sequencing depth (see Supplementary methods, Supplementary Figure S3). We also identified TFCRs using motifs from JASPAR (36) for each species. About 98% of TFCRs identified using JASPAR were overlapped with those identified using CIS-BP (Supplementary Table S5. Here, TFCRs that share over 50% base pairs are defined as overlapped). These results show that the identification of TFCRs using CIS-BP is robust.

Shuffled TFCRs and TFBSs were generated by the shuffle subcommand of BEDTools (37). Stable TFCRs were identified by 1bp overlapping between consecutive stages. Gained TFCRs were compared to the previous stage by 1bp overlapping. Lost TFCRs were compared to the next stage by 1bp overlapping.

### Analysis of the evolution of TFCRs by defining top and bottom TFCRs among species

To evaluate the regulatory evolution of TFCRs, we defined the top and bottom TFCRs. The TFCRs of each species were sorted from high to low complexity. The first 50 TFCRs in yeast were selected as top TFCRs and the last 50 were bottom TFCRs. In other species, the first 50 × N TFCRs and the last 50 × N TFCRs were selected as top TFCRs and bottom TFCRs respectively according to the multiple N of their genome size to yeast. TFCR was associated with a gene when the TFCR and gene promoter regions overlapped. The gene promoter was defined as the upstream and downstream 2kb region of the transcription start site.

### Synchronization assessment for TFCR complexity and gene expression

To evaluate the regulatory potential of TFCR on gene expression, we defined RegulatoryScore to measure the synchronization between TFCR complexity and gene expression. First, we divided TFCR and gene expression equally into 10 groups according to their complexity or expression from low to high and got the group number of each TFCR and gene expression. Next, the ChIPseeker package was used to annotate the relative position of TFCRs and genes. Gene annotation files were downloaded from Ensemble. The gene promoter was defined as the upstream and downstream 2 kb region of the transcription start site. According to the relative position of the TFCR and the gene in the genome, each TFCR was associated with the nearest gene's promoter. Finally, the following formula is used to calculate the synchronization between TFCR complexity and gene expression.


\begin{eqnarray*}regulatoryScore = \frac{{{G}_{tfcr}}}{9} \cdot \frac{{{G}_{expr}}}{9} \cdot {e}^{ - \frac{{\left| {{G}_{tfcr} - {G}_{expr}} \right|}}{5}}\end{eqnarray*}

where *G*_tfcr_ represents the group number of TFCR complexity and *G*_expr_ represents the group number of gene expressions. RegulatoryScore is a number between 0 and 1, with a higher value indicating the better synchronization between the TFCR complexity and gene expression. The good synchronization means that when the difference between the TFCR group and gene expression group is smaller, gene with high complexity TFCR shows high expression. When RegulatoryScore > 0.3, the complexity of gene expression and promoter-associated TFCR are relatively high (groups 5–9). According to the ranking of RegulatoryScore, the higher-ranked genes have better synchronization of their TFCR complexity and gene expression. The robustness of RegulatoryScore was evaluated using downsampled ATAC-seq data of hESC under different sequencing depths (see Supplementary methods, Supplementary Figure S4).

### Functional analysis of species-specific TFCR

The genes with the top 20% RegulatoryScore were considered as the ones with good synchronization of TFCR complexity and gene expression. Species-specific genes were defined as genes that exhibited good synchronization only in one species. Among all homologous genes, we first kept the gene set that ranked the top 20% in RegulatoryScore for each species, then we selected the genes that are present only once in above gene sets as the species-specific genes. Otherwise, if a gene is present in the top 20% for more than one species, it will not be included as species-specific. Highly conserved genes were defined as genes that exhibited good synchronization in all species. Gene ontology analysis was performed using the clusterProfiler package (38), with significance thresholds set at *P*-value <0.05 and *q*-value <0.2. In order to compare the enriched GO terms between species, we converted the genes of other species into human homologues genes and then annotated it with GO terms from human.

### Explainable machine learning models for RegulatoryScore prediction

For RegulatoryScore prediction, we employed eleven different machine learning-based methods, including CatBoost (39), Support Vector Regression (SVR) (40), Multi-layer Perceptron (MLP) (41), K-Nearest Neighbors (KNN) (42), Decision Tree (DT) (43), Random Forest (RF) (44), Adaptive Boosting (AdaBoost) (45), Gradient Boosting Decision Tree (GBDT) (46), eXtreme Gradient Boosting (XGBoost) (47), Light Gradient Boosting Machine (LightGBM) (48) and Convolutional Neural Network (CNN) (49). The input features of these methods included the promoter sequence, TFCR sequence and the distance between TFCR and promoter boundary. The sequence features were one-hot encoded (50). Each nucleotide (A, C, G, T) was represented by a binary vector of length four, with a value of 1 at the position corresponding to the nucleotide and 0 at all other positions. The maximum length of all promoter sequences and TFCR sequences was 4000 nt and 11113 nt, respectively, and we padded sequences shorter than the maximum length with zeros to the maximum length. Dataset of each species and each stage was randomly split into training and test subsets with the proportions of 80% and 20%, respectively. Hyperparameter tuning of models was achieved through a combination of manual tuning and grid search methodologies. We performed 5-fold cross-validation on the stage-specific and species-specific training set for model selection and got 407 different stage-specific and species-specific models in total (11 different methods × 37 different stages and species). Then, all the models were evaluated on the corresponding test set based on the Pearson correlation coefficient (*r*), Spearman correlation coefficient (*R*), Coefficient of determination (*R*^2^), mean absolute error (MAE) and root mean squared error (RMSE) between the predicted and practical RegulatoryScore. The Pearson correlation coefficient (*r*) was utilized to assess the strength and direction of linear relationships, while the Spearman correlation coefficient (*R*) provided insights into the monotonic relationship. The Coefficient of determination (*R*^2^) helped in understanding how well the model explained the variability of the data. Furthermore, the mean absolute error (MAE) was calculated to determine the average absolute differences between predicted and actual values, whereas the root mean squared error (RMSE) was employed to penalize larger errors more heavily.

To predict and interpret RegulatoryScore across various species and stages universally with a unique model rather than numerous models, we first constructed a large pooled training set. This data set was constructed by merging 30 000 training samples from each of six species, and the 30 000 samples of each species were resampled from all stages of that species. Because the stage number N of each species was different with a range of 1–14, we resampled $\frac{{30\,\,000}}{N}$ training samples with replacement from the separate training set of each stage and merged together. Therefore, the pooled training set contained 180 000 training samples and covered all species and stages. We retrained a CatBoost model on the pooled training set with the hyperparameter combination of loss_function = RMSE, iterations = 2000, learning_rate = 0.1, l2_leaf_reg = 5, depth = 12, border_count = 128, bootstrap_type = Bernoulli and boosting_type = Ordered, and then evaluated the CatBoost model on all separate test set of all stages and species.

The Tree SHAP (SHapley Additive exPlanations) method is a game theoretic approach to explain the output of a machine learning model (51,52). In the Tree SHAP method, a per-sample importance score named SHAP value is assigned to each feature from the CatBoost model. The SHAP value, which represents the contribution of the feature on the base value to the model output, was computed on the basis of a game theoretic Shapley value for optimal credit allocation, and the sum of all SHAP values and the base value of a sample equals its RegulatoryScore. We ranked the input features based on the mean absolute SHAP value and showed SHAP value distributions for the entire dataset of all species and stages in our CatBoost model. PredictionValuesChange value shows how much on average the prediction changes if the feature value changes, and LossFunctionChange value represents the difference between the loss value of the model with this feature and without it.

### Comparison of SEs and TFCR9

SEs annotation of hESC was downloaded from SEdb (53). To compare the overlap region between SEs and TFCRs, TFCRs within 20 000 bp were merged by BEDTools (37). Essential genes were collected from Wang *et al.* (54), which encode components of fundamental pathways and required for proliferation and survival. Genes activated at ZGA were collected from Fan *et al.* (55). Housekeeping genes were downloaded from Eisenberg *et al.* (56). Evolutionarily old and young genes were downloaded from Neme *et al.* (57). Cancer-related genes were collected from previous studies (58,59). Enrichment analysis in different gene sets was conducted by hypergeometric tests.

## Results

### Identification and characterization of TFCRs

**Figure 1.**
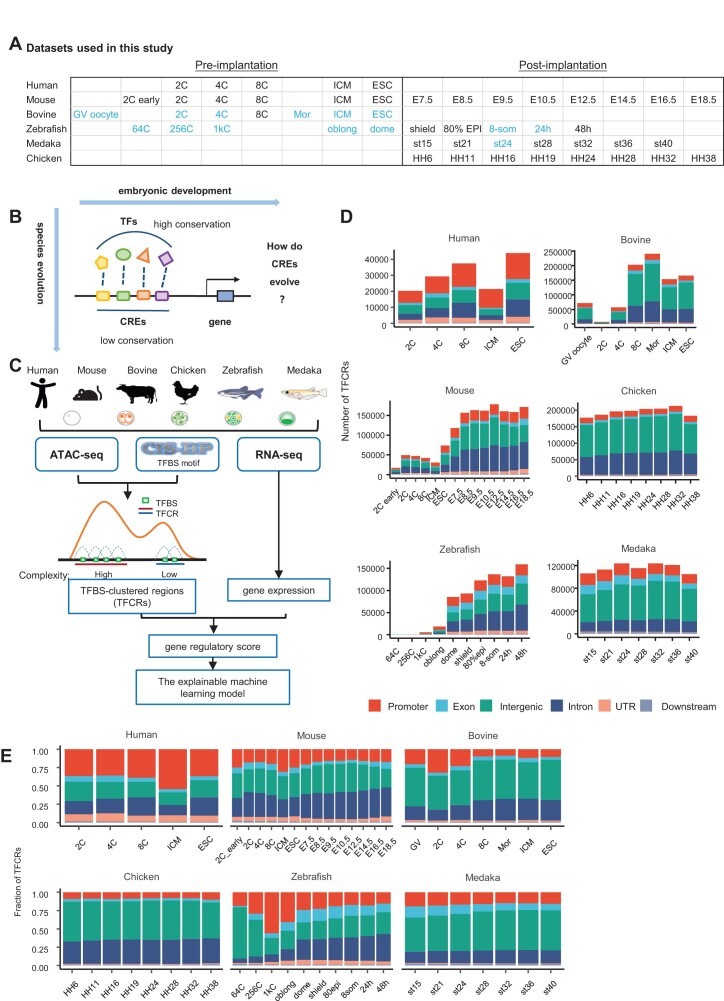
Data quality control and TFCR analysis workflow. (**A**) Species and stages that collected ATAC-seq and RNA-seq data covered. The blue color means that there is no RNA-seq data at this stage. (**B**) A schematic diagram of how sequences evolve to regulate gene expression. (**C**) An overview of TFCRs analysis workflow. ATAC-seq and CIS-BP database were used to identify TFCRs. Gene expression was obtained from RNA-seq. RegulatoryScore was calculated to evaluate the synchronization between TFCR complexity and gene expression. By comparison, species-specific and highly-conserved TFCRs were recognized for further functional analysis. Species-specific TFCRs means those with top 20% RegulatoryScore in only one specie and low RegulatoryScore in other species. (**D**) The number of TFCRs identified in each stage of species. The stacked color bar represents the number of TFCRs located in each functional element in genome. Promoter is defined as +2 kb/–2 kb region of transcription start site of gene. (**E**) The genomic distribution of TFCRs identified in each stage of species.

We collected the open chromatin data of human, mouse, bovine, chicken, zebrafish and medaka from previous studies (7–10,19,20,23,24) (Figure 1A). These data covered the early and late stages of embryonic development. Because the development time between species is difficult to synchronize well, we select relatively similar developmental stages of each species to compare the regulatory patterns among species (i.e. ESC stage in human and mouse, 8-cell stage in bovine, HH6 stage in chicken, shield stage in zebrafish, and stage15 in medaka). The 8-cell stage was selected in bovine because no gene expression data were available for the ESC stage.

Many transcription factors are highly conserved during evolution. However, it is not fully understood how sequences evolve to regulate genes between species during embryonic development (Figure 1B). Here, we identified the TFCRs of each species at each stage in a similar way as described in a previous study (17) (Figure 1C, Supplementary Table S6, see Materials and methods). TFCRs had the lower percentage of genome coverage (0.7–1.6%) in human and mouse relative to other species (Supplementary Figure S5A). The number of identified TFCRs in human was similar to that of mouse before mESC stage, both <50 000 (Figure 1D). Most human TFCRs were distributed near promoters (37%), while most mouse TFCRs located in intergenic and introns regions (34%). In bovine, chicken, zebrafish, and medaka, most TFCRs located in intergenic regions (Figure 1E). To gauge bias from sequencing depth, the randomly subsampled ATAC-seq peak sets of human, mouse, and bovine from a recent paper were employed (9). TFCRs were identified from those subsampled peaks, which were called from about 30 million uniquely mapped reads for those three species. The genome coverage of TFCRs in human and mouse was still lower than that in bovine (Supplementary Figure S5B). After annotating the genomic region of those TFCRs, the subsampled results were consistent with our previous results. In human, more TFCRs located in promoters compared to mouse and bovine (Supplementary Figure S5C, D). The identified TFCRs had a higher proportion of promoters than randomly shuffled TFCRs across all species, with human showing prominent enrichment of TFCRs in promoters than the other species (Supplementary Figure S6A). Thus, the results suggested that human TFCRs, although with low coverage in the genome, were densely distributed in the promoter regions of genes and had a higher regulatory potential than other species. Chicken TFCRs, although with high coverage in the genome, were mostly in the intergenic regions. These results indicated more proximal regulatory in human than non-human mammal, chicken and fish.

The complexity of a TFCR was determined by the number of TFBSs within it. To describe TFCRs, we divided TFCRs into 10 equal groups according to their complexity (from low to high is labeled as TFCR0 to TFCR9). For most species, TFCRs with higher complexity were often located in gene promoters (Supplementary Figure S7), with TFCR9 reaching 85.1% in human promoters at ICM. This result indicated a higher complexity of transcriptional regulation near gene promoters. However, these results were not observed in the early stage in zebrafish (64-cell∼oblong). There was no obvious pattern in the distribution of TFCRs on the zebrafish genome at this developmental stage, which may indicate more stochasticity in the transcriptional regulation in zebrafish at early embryonic developmental stage.

### Stable TFCRs during embryonic development have higher complexity and are more often located in promoters

**Figure 2.**
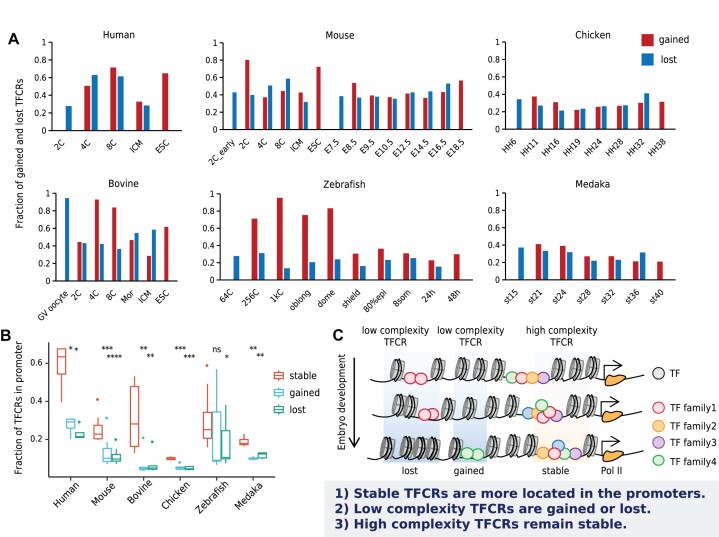
Dynamic changes of TFCRs during embryonic development. (**A**) The number of gained and lost TFCRs in each stage compared to the consecutive stages. Gained TFCRs were compared to the previous stage. Lost TFCRs were compared to the next stage. (**B**) Fraction of TFCRs located in promoters. Statistical significance is evaluated using Wilcoxon test, ****P < 0.0001, ***P < 0.001, **P < 0.01, *P < 0.05, ns *P* ≥ 0.05. The fraction of stable TFCRs in promoters were compared to that of gained and lost TFCRs, respectively. (**C**) Dynamic and stable TFCRs on the genome. All circles with different colors refer to transcription factors, and different colors refer to different TF families. When calculating the complexity of TFCRs, motif instances located in overlapping positions were combined if they belonged to the same TF family.

Extensive chromatin reorganization occurs during embryonic development (7,60). How TFCRs change with embryonic development is still not clear. In order to describe the changes of TFCRs in the early embryonic development stages of each species, we identified the gained and lost TFCRs between consecutive developmental stages. To eliminate the deviation of the identification numbers at each stage caused by sequencing depth, we compared the proportion of gained or lost TFCRs to the total number of TFCRs identified at that stage. Gained TFCRs were as high as 70–80% at the zygotic gene activation (ZGA) stage (human: 8-cell, mouse: 2-cell, bovine: 8-cell, zebrafish: 1k-cell), while only 20–56% were gained at the other stages of embryonic development (Figure 2A), indicating that human, bovine, mouse, and zebrafish activate extensive new transcriptional regulation in the ZGA stage. Moreover, gene ontology analysis revealed that ZGA-gained TFCRs associated with genes preferentially involved in several biological processes, including histone modification and covalent chromatin modification for human, mouse, and zebrafish, embryonic development for human, mouse, bovine and zebrafish, and cell fate commitment for bovine and zebrafish (Supplementary Figure S8). The embryonic development is a common event among human, mouse, bovine and zebrafish, which represents the progression of the embryo from formation of the zygote to birth. But there may still be some different molecular mechanisms and regulatory pathways based on the distinct needs of fast or slow dividing species (61). Covalent chromatin modification is central to the regulation of chromatin dynamics (62). Drastic chromatin reorganization and reprogramming of histone modification occur during ZGA (7–11). Cell fate commitment occurs during early embryonic development (60). ZGA-gained TFCRs provide the opportunity for regulatory elements to instruct the specific cell fates commitment during early embryonic development.

Next, we analyzed the complexity of dynamic TFCRs during early embryonic development among species and found that most of the gained and lost TFCRs during development were of low complexity (Supplementary Figure S9), suggesting that high complexity TFCRs were more stable during development. Moreover, the proportion of stable TFCRs located in promoters was higher than that of dynamic TFCRs (Figure 2B, Permutation test *P*-value < 0.0001). This common feature for mammal, chicken and fish showed that the high complexity TFCRs near the promoter remained relatively stable, while the low complexity TFCRs outside the promoter region was more in the process of gaining or losing. In addition, the distance between TFCRs and transcription start sites of genes decreased and the complexity of TFCRs increased during embryonic development (Supplementary Figure S10). These results indicated that transcriptional regulation is becoming more accurate and robust during embryonic development (Figure 2C).

### The ability of TFCRs to regulate genes has become increasingly complex through evolution

**Figure 3.**
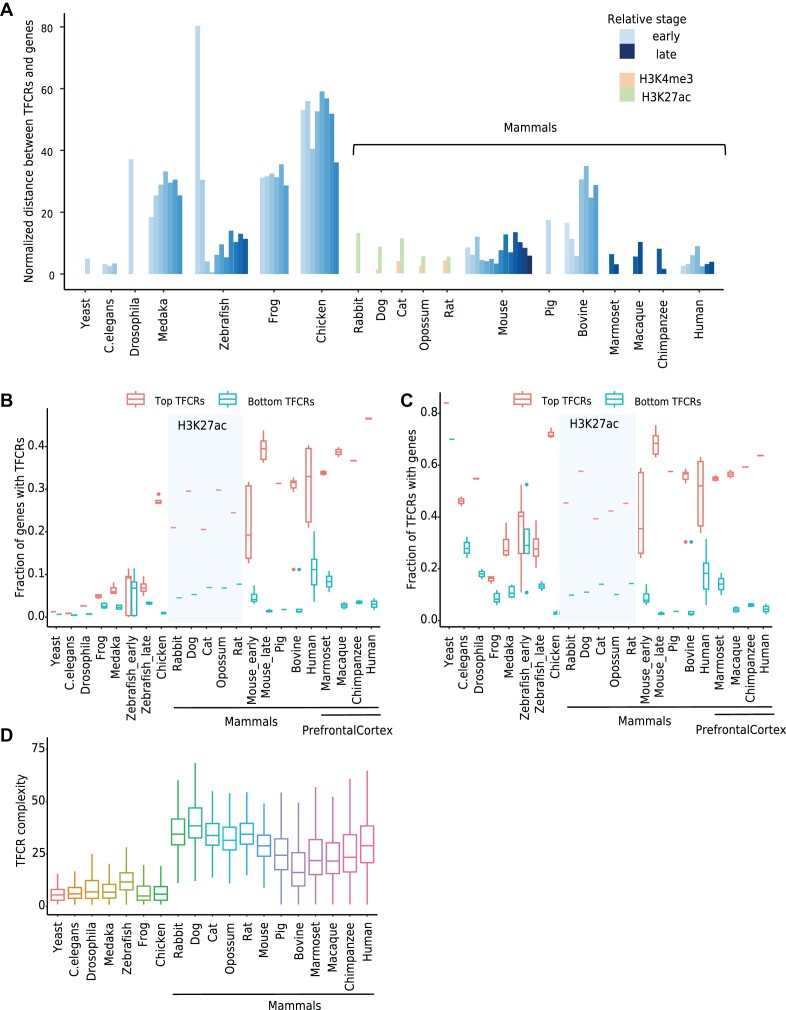
The characteristic of TFCRs among species. (**A**) The normalized distance between TFCRs and its nearest genes among different species. The color from light to dark represents the development of embryo stages. The normalized distance refers to the peak value of the distance density distribution and it then is normalized by genome size of yeast. (**B**) Fraction of genes associated with TFCRs. Top and bottom TFCRs are based on the 10% and 90% quantiles of TFCRs complexity. The TFCRs of rabbit, cat, dog, opossum and rat were identified from ChIP-seq data of H3K27ac, and the other TFCRs were identified from ATAC-seq. (**C**) Fraction of TFCRs associated with genes. (**D**) The TFCR complexity from yeast to mammals.

To explore the regulatory evolution of TFCRs, we identified TFCRs from yeast to mammals and characterized the genomic distribution of TFCRs among species. The TFCRs of rabbit, cat, dog, opossum, and rat were identified from ChIP-seq data of H3K4me3 and H3K27ac, and the other TFCRs were identified from ATAC-seq. We found that TFCRs in primates located closer to genes than those in other species (Figure 3A and Supplementary Figure S11A). To eliminate the bias of sequence depth and genome size among species, we selected top TFCRs (most complicated TFCRs, see Materials and methods) and bottom TFCRs (simplest TFCRs) to compare their relation with genes. From yeast to mammals, the fraction of genes related to top TFCRs increased, but those related to bottom TFCRs did not change a lot (Figure 3B). Also, the difference in the fraction of top TFCRs and bottom TFCRs associated with genes became greater in chicken and mammals than the other species (Figure 3C). In H3K4me3-TFCRs, the difference between the top TFCRs and bottom TFCRs associated gene ratios is small because the promoters are all located close to the genes (Supplementary Figure S11B, C). More genes were related to top TFCRs in complex organisms than in simple organisms, and the TFCR complexity in mammals was higher than that in yeast, *C. elegans* and Drosophila (Figure 3D). It indicated the accumulation of high-complexity TFCRs during evolution.

Relatively old genes were those that originated before vertebrate divergence (57). Using previous gene classification (57,63), we compared the relationship between TFCR complexity and gene age in human, mouse and zebrafish at an embryonic stage. The TFCR complexity of the promoter region of old genes was significantly higher than that of young genes (Wilcoxon rank-sum test *P*-value < 0.0001), indicating that old genes had more complex transcriptional regulation patterns (Supplementary Figure S12A). Furthermore, categories of gene ages for other species were obtained based on their homology to human. It was observed that old genes had more complex TFCRs than young genes in chicken, bovine, zebrafish, and medaka, suggesting that TFCRs may have played a role before the origin of vertebrates (Supplementary Figure S12B).

### RegulatoryScore evaluates synchronization of TFCR complexity and gene expression

**Figure 4.**
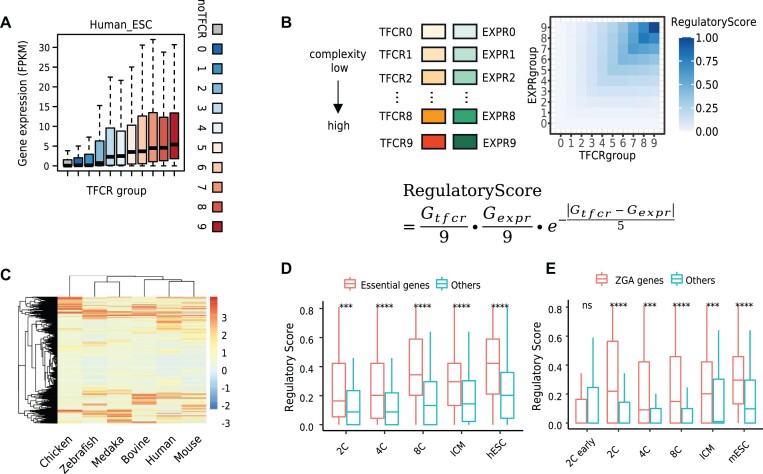
Evaluation of the regulatory relationship between TFCR and gene expression. (**A**) Expression level of genes associated with different complexity TFCRs. The grey color means that there is no TFCR located in genes’ promoter. The color from blue to red means a gradual increase of TFCR complexity. (**B**) The calculation of RegulatoryScore to measure the synchronization between TFCR complexity and gene expression. (**C**) Hierarchical clustering based on RegulatoryScore of human-ESC, mouse-ESC, bovine-8C, chicken-HH6, zebrafish-shield, medaka-stage15. The 8-cell stage was selected in bovine because no gene expression data were available for the ESC stage. The distance is defined as 1-Spearman correlation of RegulatoryScore. The hierarchical clustering was performed by hclust function with complete method in R. (**D**) The RegulatoryScore between human essential genes and others during human embryonic development. Essential genes are from Wang *et al.* Statistical significance is evaluated using Wilcoxon test, ****P  < 0.0001, ***P < 0.001, **P < 0.01, *P  < 0.05, ns *P* ≥ 0.05. (**E**) The RegulatoryScore of ZGA related genes across mouse embryonic development. Statistical significance is evaluated using Wilcoxon test, ****P < 0.0001, ***P < 0.001, **P < 0.01, *P < 0.05, ns *P* ≥ 0.05.

TFCR is the aggregation of multiple transcription factor binding sites, and its complexity is determined by the number of transcription factors within it. To explore the relationship between the complexity of TFCRs and gene expression, we associated each TFCR with its nearest gene by ChIPseeker (64). For all species, the gene expression of promoter region without TFCR association was lower than that of promoter region associated with high complexity TFCR (Figure 4A and Supplementary Figure S13). With the increase of the complexity of TFCR, the expression of its associated genes also showed a weak upward trend. To what degree the complexity of TFCR is associated with gene expression has not been fully studied.

To compare the regulatory pattern among species, 8753 homologous genes in all six species and their associated TFCRs were selected for analysis. We defined RegulatoryScore to measure the synchronization of TFCR complexity and gene expression (Figure 4B, Materials and methods). RegulatoryScore is a number between 0 and 1, with a higher value indicating the better synchronization between the TFCR complexity and gene expression. It quantitatively reflected the potential regulatory capacity of TFCRs to gene expression. Genes at similar developmental stages of species clustered by RegulatoryScore roughly reflected the evolutionary relationship between species (Figure 4C). Since the genomic distribution of ATAC-seq peaks and TFCRs in chicken is different from those in other species (Figure 1D), it's not surprising that the RegulatoryScore in chicken is more diverged than other species. The proportion of ATAC-seq peaks and TFCRs in the promoter in chicken is lower than that in other species (Supplementary Figure S6B). The proportion of identified TFCRs and randomly shuffled TFCRs in the promoter region is similar in chicken (Supplementary Figure S6A), indicating that the regulatory model of chicken may be different from that of other species. Since both mouse and human data were taken from the ESC stage, whereas bovine data were taken from the 8-cell stage, the relationship between human and mouse was closer than that between human and bovine. These results demonstrated the accuracy of the RegulatoryScore in reflecting the regulatory pattern of TFCR complexity and gene expression.

Next, we investigated the RegulatoryScore values in different gene sets. Essential genes encode components of fundamental pathways (54) and are more evolutionarily conserved than non-essential genes (65). RegulatoryScore of essential genes in human was higher than that of randomly selected non-essential genes (Figure 4C), indicating that TFCR may have an important role in regulating these essential genes. In addition, the RegulatoryScore of ZGA genes during mouse early embryonic development were significantly higher than that of the other genes from 2-cell stage to mESC stage (Wilcoxon rank-sum test *P*-value <0.05, Figure 4D). This result coincided with the phenomenon of mouse zygote gene activation at the 2-cell stage, indicating that the ZGA gene had its unique regulatory characteristics from the 2-cell stage. These results showed that RegulatoryScore was a good index to characterize the biological processes from the complexity of TFCRs and gene expression among species.

### Genes with high RegulatoryScore show species-specific biological processes

Next, we selected the similar developmental stages of the six species and compared the sharing of genes with high RegulatoryScore among species. For each species, we selected the top 20% of RegulatoryScore ranked genes as well-coordinated genes, which had good synchronization of TFCR complexity and gene expression. Functional analysis of these well-coordinated species-specific genes also showed species specificity in biological process (Supplementary Figure S14A). Sulfur compound metabolic processes were enriched in chicken-specific genes (Supplementary Figure S14B), suggesting that a sulfur compound metabolic pathway in chicken may differ from mammals and fish, which is consistent with a cysteine lyase associated sulfur compound metabolism recently reported to be present in chicken embryos, but not in mammals (66). In addition, fish-conserved genes were enriched in pigment metabolic processes (Supplementary Figure S14C). As widely used model animals, zebrafish and medaka have more pigment cell types than mammals (67). This result suggested a different pigment metabolic pattern in zebrafish and medaka than in mammals and chicken.

Human-specific genes with high RegulatroyScore were mainly enriched in regulation of neuron projection development, extracellular structural organization, synaptic organization, etc. Compared to genes involved in basic biological processes (e.g. endosomal system organization, regulation of mRNA stability), genes related to neuronal projection development or synaptic organization showed significantly higher RegulatoryScore in human embryos than in other species (Supplementary Figure S15). Previous study found some human genes were most significantly associated with neurodevelopmental phenotypes in mouse or human ESCs (68). Human evolved regulatory elements and human accelerated regions were previously reported to be associated with neurodevelopment (69,70). These results showed the specificity of human neuronal development, which suggested that TFCRs may regulate human neuron development and contribute to neural development that differs in human from non-human mammal, chicken and fish.

### Explainable machine learning models illustrate the importance of distance between TFCR and promoter for RegulatoryScore prediction

**Figure 5.**
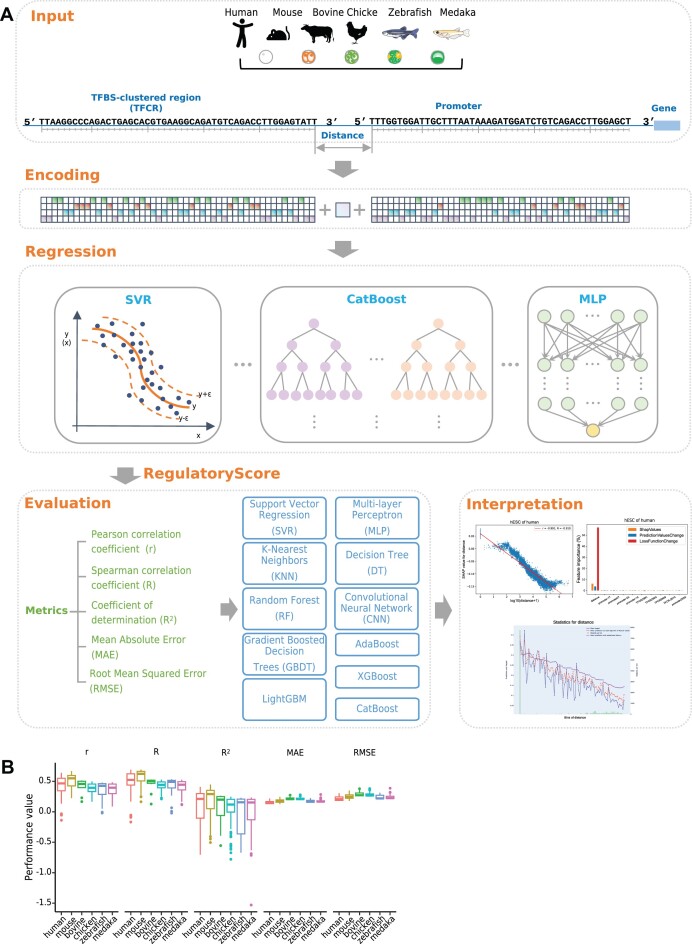
The Explainable machine learning models for RegulatoryScore prediction. (**A**) Schematic representation of the explainable machine learning models for RegulatoryScore prediction and interpretation. (**B**) Performance of the CatBoost model in training and test data.

To explore the hidden relationships and patterns between sequence features and RegulatoryScore, we employed eleven different machine learning-based methods to predict the RegulatoryScore based on the promoter sequence, TFCR sequence and the distance between them (Figure 5A). After evaluating all these models for each species and each stage, most CatBoost models outperformed the corresponding models of the other ten methods (Supplementary Figure S16, see Materials and methods). To predict and interpret RegulatoryScore without bias across various species and stages universally with a unique general model rather than numerous models, we retrained a CatBoost model on a pooled training set that was resampled from all separate training sets of all species and all stages (see Materials and methods). The CatBoost model demonstrated favorable performance, exhibiting a positive correlation and a modest prediction error when estimating the RegulatoryScore. These findings imply its promising learning capacity and reasonable generalization ability for RegulatoryScore prediction across various species and developmental stages (Figure 5B).

**Figure 6.**
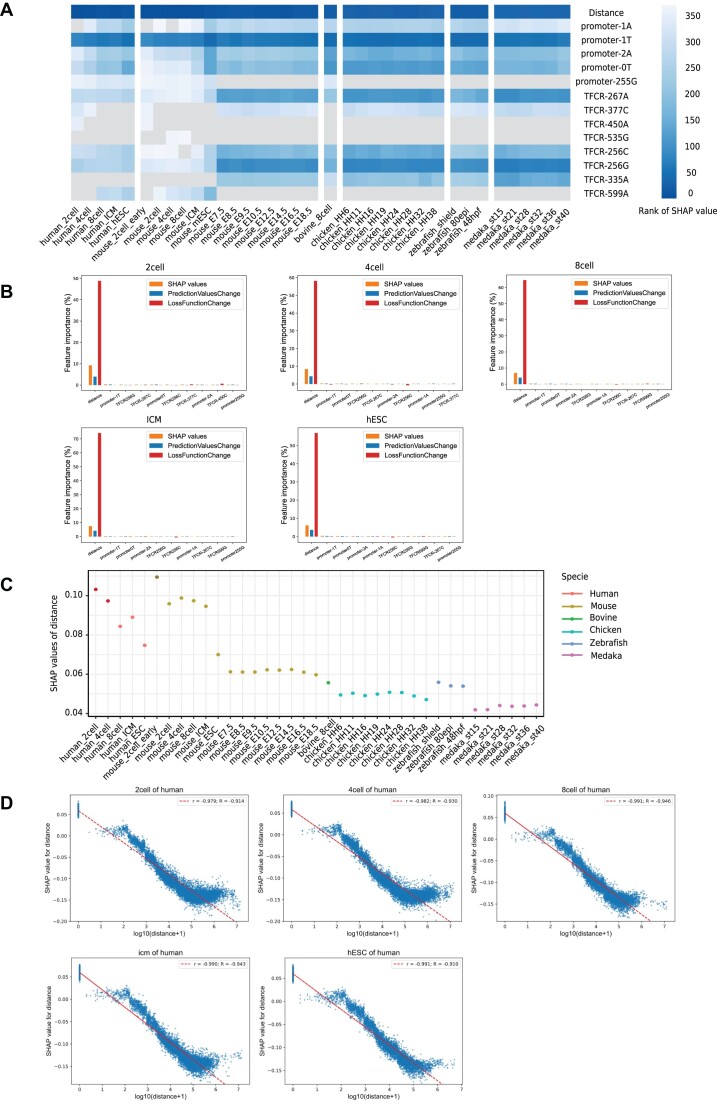
The feature importance for RegulatoryScore prediction in species. (**A**) The heatmap for the ranks of SHAP values of top 10 features among species. (**B**) The SHAP values, PredicionValuesChange, LossFunctionChange for feature importance in human. (**C**) The mean absolute value of the SHAP values of distance among species. (**D**) The relationship between SHAP values of distance and distances for each stage in human. Pearson correlation coefficient was represented as *r*. Spearman correlation coefficient was represented as *R*.

To evaluate factors associated with RegulatoryScore in a more systematic manner, we next performed Tree SHAP (SHapley Additive exPlanations merged into the CatBoost model) to explain the feature importance of the CatBoost model based on all input features (see Methods). The SHAP value of a feature represents the contribution of the feature to the output RegulatoryScore of a sample. We calculated the mean absolute value of the SHAP values for each feature over all samples, which represents the feature importance for the CatBoost model. When high feature values were linked with high and low RegulatoryScore, then the features could be classified as favored and disfavored features, respectively. We ranked the feature importance values of all input features and selected the top 10 features of all species and all stages. Notably, distance emerged as the most important feature (disfavored feature) for RegulatoryScore prediction across all species and all stages, and increasing the distance would decrease the RegulatoryScore (Figure 6A and Supplementary Figure S17). T at one base upstream of the transcription start site was also important for the prediction of RegulatoryScore in all species (disfavored feature, Figure 6A and Supplementary Figure S17), which meant T at one base upstream of the transcription start site was linked with low RegulatoryScore. If thymine at this specified position was less favorable for the binding of a critical transcription factor associated with the regulation of TFCRs, it could result in decreased binding affinity of transcription factors. This reduced affinity might lead to a decrease in the regulatory score.

Besides, PredictionValuesChange and LossFunctionChange methods also showed the distance as the most important feature across all species and all stages (Figure 6B and Supplementary Figure S18), which is in line with the SHAP method-based feature importance.

Then we compared the mean absolute value of the SHAP values of the distance across all species and all stages and found that the effect of distance on RegulatoryScore prediction was higher in early embryonic than that in late stage (Figure 6C). Especially before the ZGA in human and mice (human 2 cells, human 4 cells and mouse 2 cells early), the importance of distance to the prediction of RegulatoryScore was higher than that after ZGA. This suggested that the distance between TFCR and gene promoter before ZGA was more important to the regulation of gene expression by TFCR, while other factors may be involved in the regulation of gene expression by TFCR after ZGA.

To understand how distance feature effects the output RegulatoryScore of the CatBoost model, we plotted the SHAP value of distance versus distance for all samples. Since SHAP values of distance represent of distance's responsibility for a change in RegulatoryScore predicted by the model, these results showed that the predicted RegulatoryScore decreased as the distance increased and there was significant negative correlation between them in all species and all stages (Figure 6D and Supplementary Figure S19). The larger the distance between TFCR and promoter was, the smaller the RegulatoryScore was (Supplemantary files). These results showed that the smaller distance was more contributed for predicting the RegulatoryScore. The CatBoost model revealed the importance of distance between TFCR and promoter for regulatory models in these species.

## Discussion

CREs, including promoters, enhancers, and silencers, have a crucial role in regulating gene expression. The uneven distribution of CREs across the genome may indicate the different degrees of transcription activity in different genomic regions. Similar to the definition of TFCRs, SE is a cluster of enhancers in close genomic proximity. Enhancers and SEs regulate gene expression distally, which can determine cell identity (71) and lineage specificity (72–74). However, the differences between TFCRs and SEs have not been described in detail. We compared TFCRs to SEs in hESC, and found that the number of SEs was lower than that of TFCRs, and the genomic size of SEs was larger than that of TFCRs (Supplementary Figure S20A). In hESC, 56% of enhancers overlapped with TFCRs, 31% SEs located at the most complicated TFCR group TFCR9, and 66% of SEs had TFCRs (Supplementary Figure S20B). A possible explanation for this high overlap ratio is that TFCRs plays a key role in SEs. Because of their short length, TFCRs may be more accurate in locating functional sequences than SEs. We also found that both SE and TFCR9 were enriched in housekeeping genes and cancer-related genes (Supplementary Figure S20C). Nevertheless, the proportion of cancer genes in SE was higher than that in TFCR9, and the proportion of housekeeping genes in TFCR9 was higher than that in SE. In addition, SE enriched essential genes, while TFCR9 enriched relatively old genes, indicating different biological functions between SE and TFCRs.

Herein, we identified TFCRs with complexity scores at embryo stages from fish, chicken, and mammals, and proposed the regulation model of TFCRs among species.

**Figure 7.**
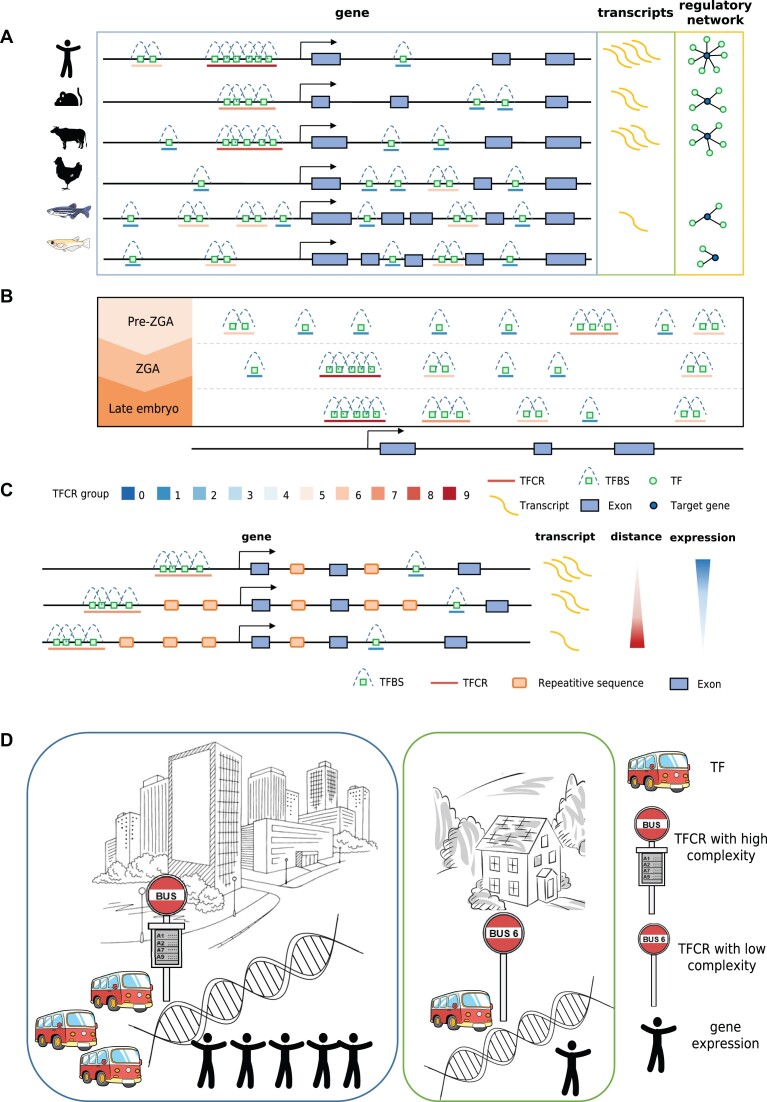
The regulatory pattern of TFCRs among species. (**A**) The regulatory pattern of TFCRs among species. (**B**) The regulatory pattern of TFCRs during embryonic development. (**C**) Schematic representation of the random insertion or deletion of repetitive sequences that regulate the distance between TFCR and promoter. From top to bottom, the distance from the TFCR to the promoter increases and gene expression decreases. (**D**) A public transport system with high utilization. There are more bus lines in areas with large passenger flow, while in desolate areas there are only a few bus lines.

We characterized the nature of TFCRs during embryonic development in different species (Figure 7A). The comparison of TFCRs in genomic distribution revealed that human TFCRs were more enriched in promoter regions compared to chicken and fish (zebrafish, medaka), suggesting that the transcriptional regulation pattern in human may prefer proximal and complicated transcriptional regulation. The enrichment of chicken and fish TFCRs in intergenic regions suggests a possible preference for distal regulation via enhancers. Higher complexity TFCRs in human means that more TFs may be involved in gene regulation, suggesting more complex regulatory patterns than that in chicken and fish. When one transcription factor fails to bind to its sites, paralogous transcription factors can still initiate the gene transcription, showing the robustness of transcriptional regulation.

With embryonic development, a common event is that stable TFCRs have higher complexity and located more in promoters than gained or lost TFCRs between stages, implying a more accurate and robust way of gene regulation among species (Figure 7B). The quantitative and complexity changes of TFCRs in various species during embryonic development suggested drastic changes in transcriptional regulation at zygote gene activation compared to later embryonic development in human, mouse, bovine, and zebrafish. Stable TFCRs during embryonic development have a higher degree of complexity, which may have more complex regulation to maintain some essential biological processes. In contrast, dynamical TFCRs have lower complexity and may be regulated by certain specific transcription factors involved exclusively at a certain stage.

From simple species to complex species, not only the sequence of CREs has changed greatly, but also the regulation patterns of TFs and CREs have changed. To find the key factors that play a role in the regulatory ability of TFCR, we evaluated several machine learning models for RegulatoryScore prediction and CatBoost outperformed the other prediction models. SHAP analysis was employed to provide additional insights into the developed CatBoost model. The explainable model revealed that distance between promoter and TFCR is the most important factor affecting TFCR-regulated gene expression among species and developmental stages.

Repetitive sequences constitute more than half of the human genome, which play a critical role in gene regulation and genome architecture. Transposition, expansion, and contraction of repetitive sequences drive genome plasticity and adaptive evolution (75,76). We hypothesized that gene expression was regulated by altering the distance of the TFCR from the promoter through the random insertion or deletion of repetitive sequences in the genome (Figure 7C).

In complex species, the distribution of CREs in the genome is more aggregated and closer to the TSSs of genes. The complexity of TFCRs near those promoters is high, indicating that the regulation near promoters is more robust. The distribution of CREs during early embryonic development is similar to the evolution of CREs among species. During embryonic development, the distribution of CREs changes from scattered, far from the TSS, to more aggregated, closer to the TSS, corresponding to the increased ability to regulate transcription.

This reminds us that the regulatory model of TFCR can be compared to urban public transport network planning. Traffic utilization is higher in the city center, while it is lower far from the city center. A good public transport system should have more bus lines in areas with larger passenger flow, while in desolate areas there are only a few bus lines to achieve efficient utilization (Figure 7D). Here, a TFCR is like a bus stop, each TF is a bus, the complex TFCRs is a bus stop with multiple lines, and the level of gene expression is analogous to the number of people on the bus. Transcriptional regulation in human is like a public transport system with high utilization. More complex TFCRs are distributed in the promoter region of the gene, which provides more opportunities for TFs binding and gene transcription. TFCRs with low complexity is mostly distributed outside the promoter. On the other hand, fish and chicken have relatively low utilization rate of transportation system, and they do not fully consider the complexity of the surrounding environment and passenger flow when setting bus stop routes. High complexity TFCRs exists in other areas in addition to promoters. Just like in a desolate place, there are many routes of buses, which will cause a waste of buses. The evolution of TFCRs from simple to complex organisms is a process of progressively improving the efficiency of transcriptional regulation, and embryonic development is a similar process.

Genome conformation is a key factor affecting transcriptional regulation (77). It was reported that COREs were enriched at topologically associated domain (TAD) boundaries and were preferentially bound by the chromatin looping factors CTCF and cohesion (18). Our previous study revealed that some TFCRs were spatially adjacent (78). These studies indicated that TFCRs also involved changes in the three-dimensional structure of the genome. Several studies reported that 3D genome conformation functions in regulating gene expression during evolution (79–81). However, due to the challenge of sampling from embryos, the Hi-C data of early embryos are few across species. In the future, as more Hi-C data become available, the mechanism of transcriptional regulation by TFCRs in early embryos across species can be analyzed in more detail.

## Supplementary Material

gkae441_Supplemental_Files

## Data Availability

The code for TFCR identification and all the results of identified TFCRs are available at https://doi.org/10.5281/zenodo.11149753.
