# Supplementary material for: The developmental and evolutionary characteristics of transcription factor binding site clustered regions based on an explainable machine learning model
Source: Nucleic Acids Res. 2024 May 30;52(13):7610–26. doi: 10.1093/nar/gkae441 (PMC11260490; doi:10.1093/nar/gkae441)
Supplement: gkae441_Supplemental_Files [file gkae441_supplemental_files.zip › Supplementary figures.pdf]

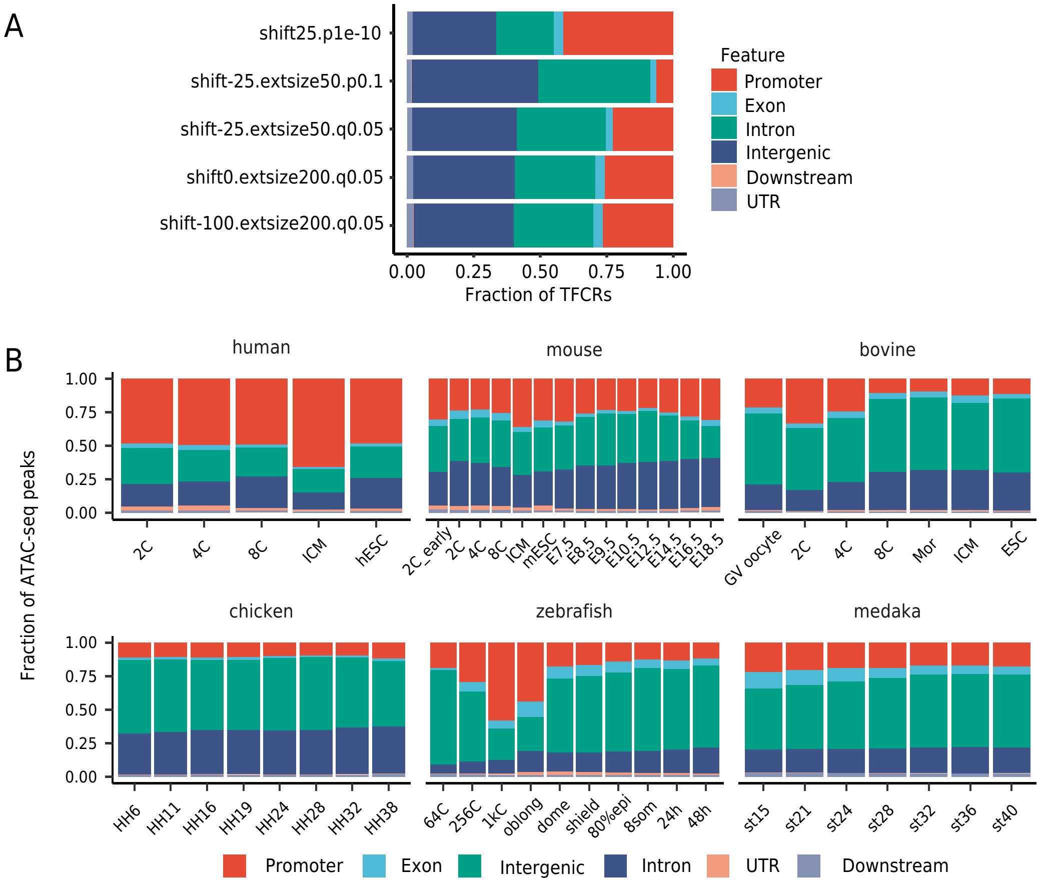

Figure S1. The genomic distribution of ATAC-seq peaks. (A) The genomic distribution of ATAC-seq peaks at hESC called by MACS2 using different parameters. (B) The genomic distribution of ATAC-seq peaks among species.

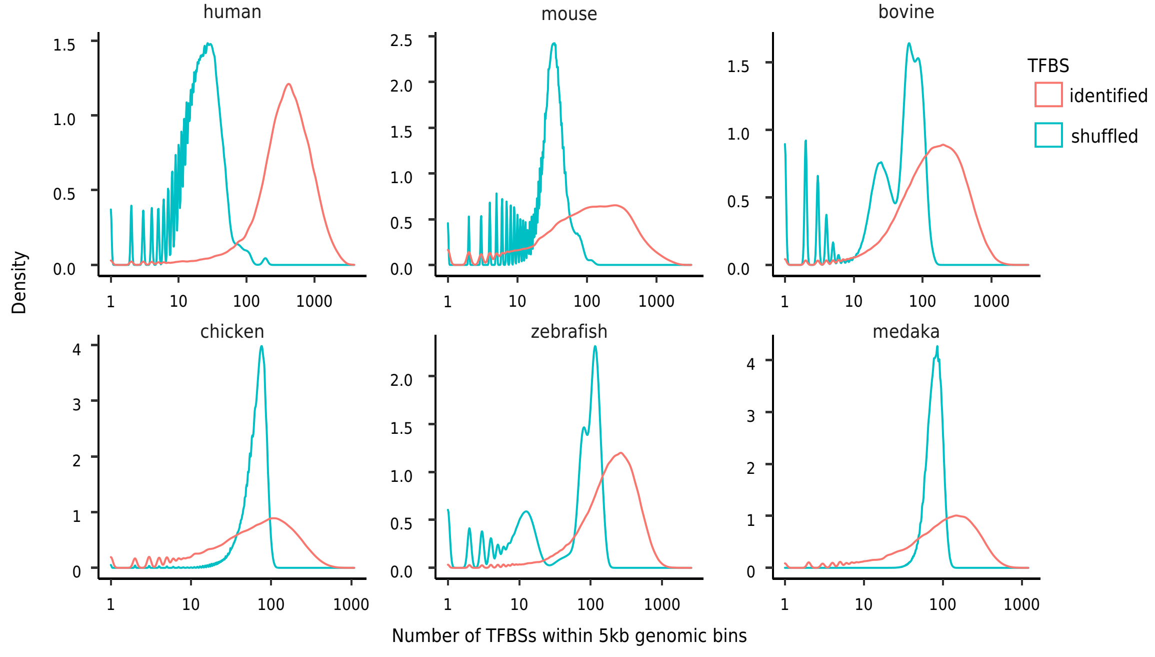

Figure S2. The density plot of the number of TFBSs located in 5kb genomic bins within each specie. Red and blue lines represents the distribution of the number of identified and randomly shuffled TFBSs within 5kb genomic bins, respectively. x axis is log10 scaled.

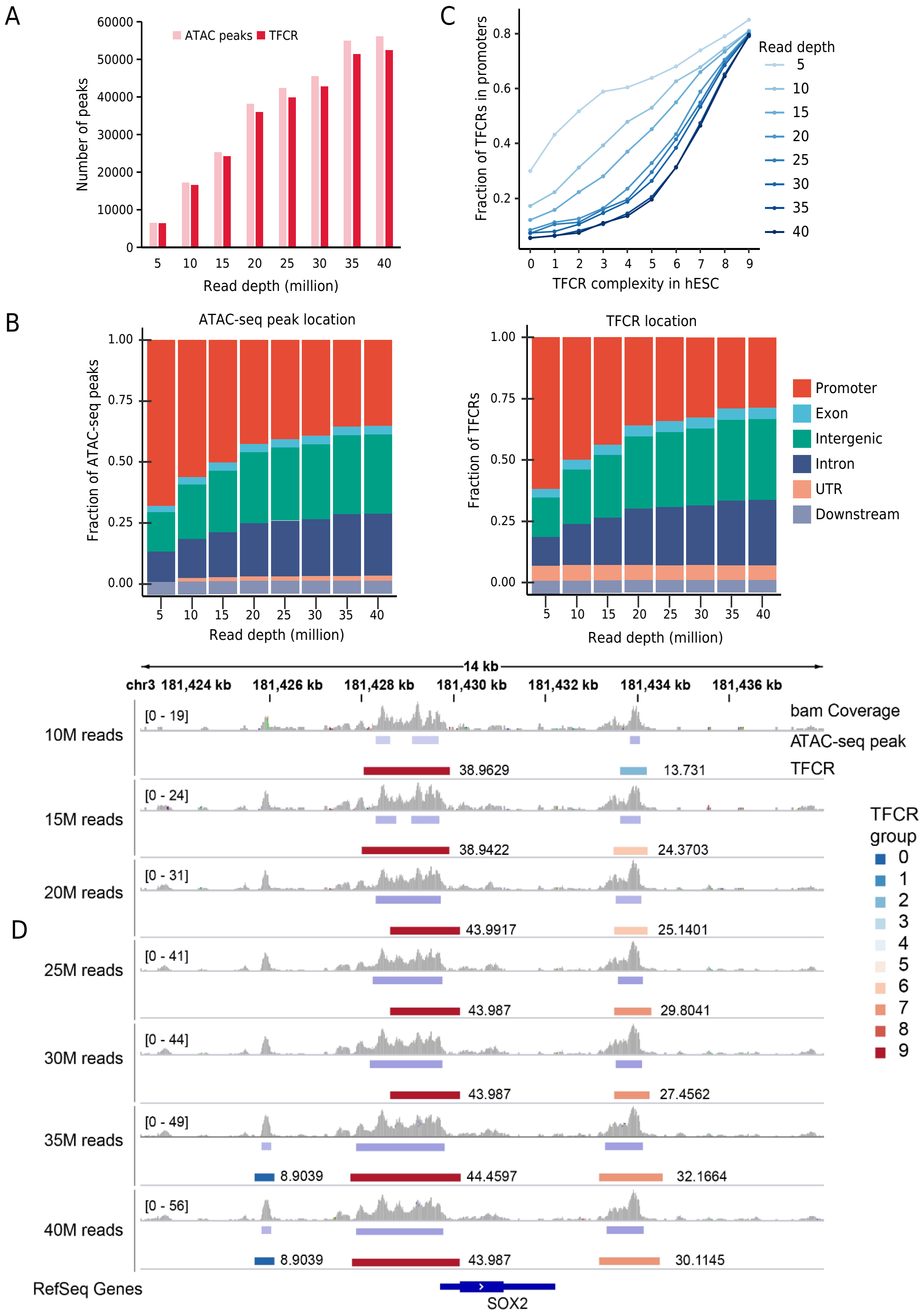

Figure S3. The robustness of TFCR identification under different sequencing depths.
(A) The number of ATAC-seq peaks and TFCRs identified from different sequencing depths. (B) The distribution of ATAC-seq peaks and TFCRs in functional elements in genome at different sequencing depths. (C) The fraction of TFCRs located in promoters at different sequencing depths. (D) The overview of TFCRs surrounding SOX2 at different sequencing depths. M: million.

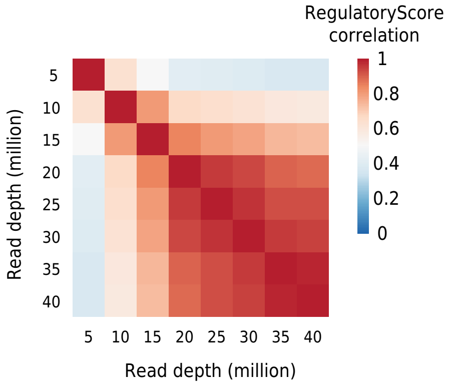

Figure S4. The Pearson correlation of RegulatoryScore calculated from different sequencing depths.

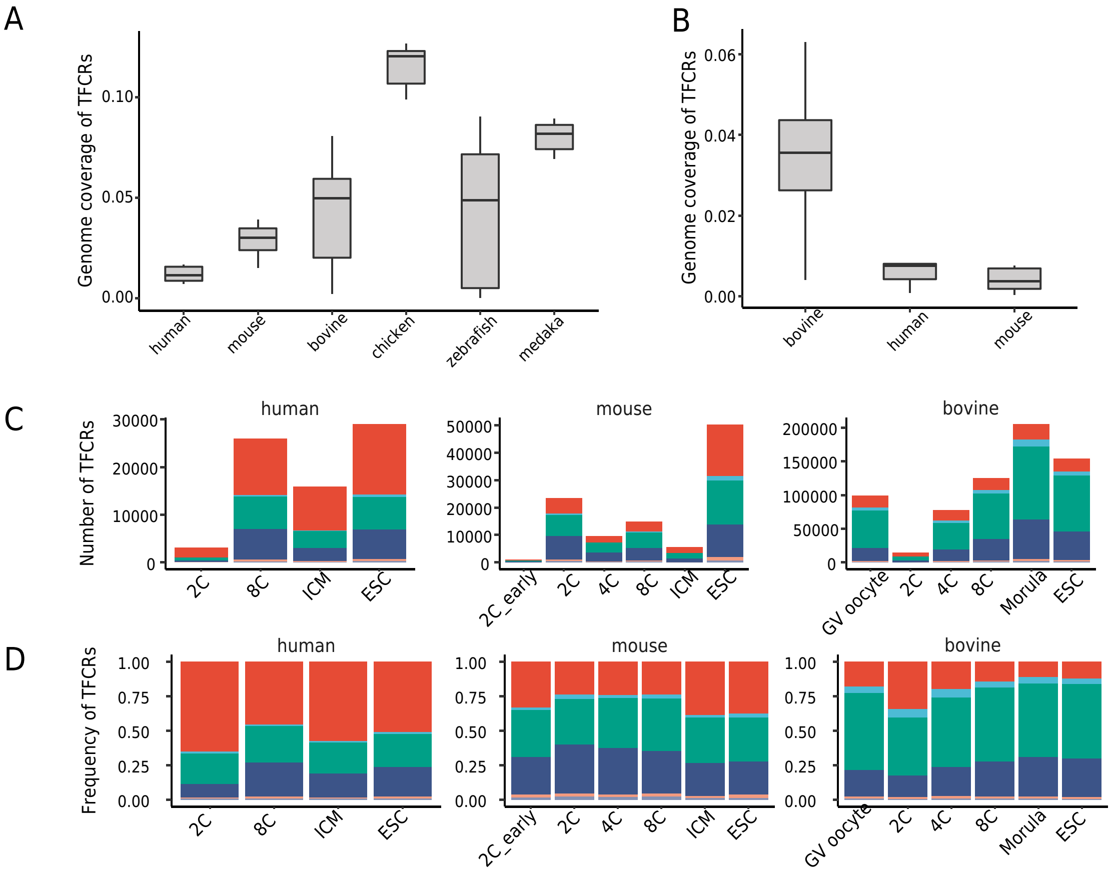

Figure S5. Genome coverage and genomic distribution of TFCRs across species.
(A) Genome coverage of identified TFCRs across species. (B) Genome coverage of TFCRs identified from subsampled 30 million ATAC-seq uniquely mapped reads from human, mouse and bovine. (C) The number of TFCRs identified from subsampled ATAC-seq peaks. ATAC-seq peak sets were called from subsampled 30 million ATAC-seq uniquely mapped reads from human,mouse and bovine. (D) The genomic distribution of TFCRs identified from subsampled ATAC-seq peak sets of human, mouse and bovine.

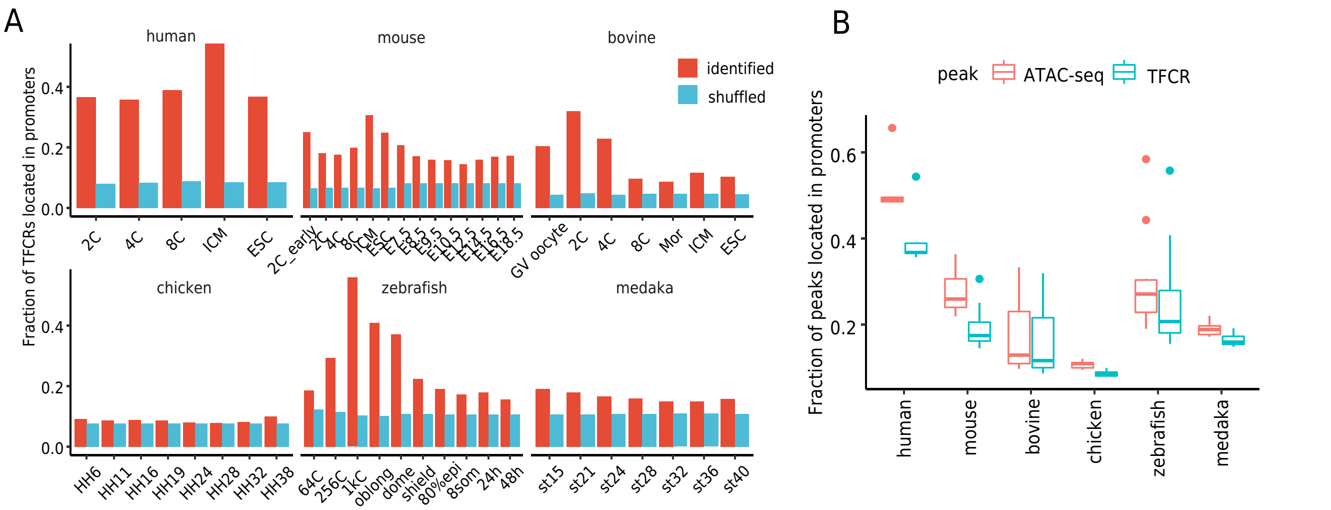

Figure S6. The fraction of ATAC-seq peaks and TFCRs in promoters. (A) The proportion of identified TFCRs and randomly shuffled TFCRs in the promoter. (B) The fraction of ATAC-seq peaks and TFCRs in promoters.

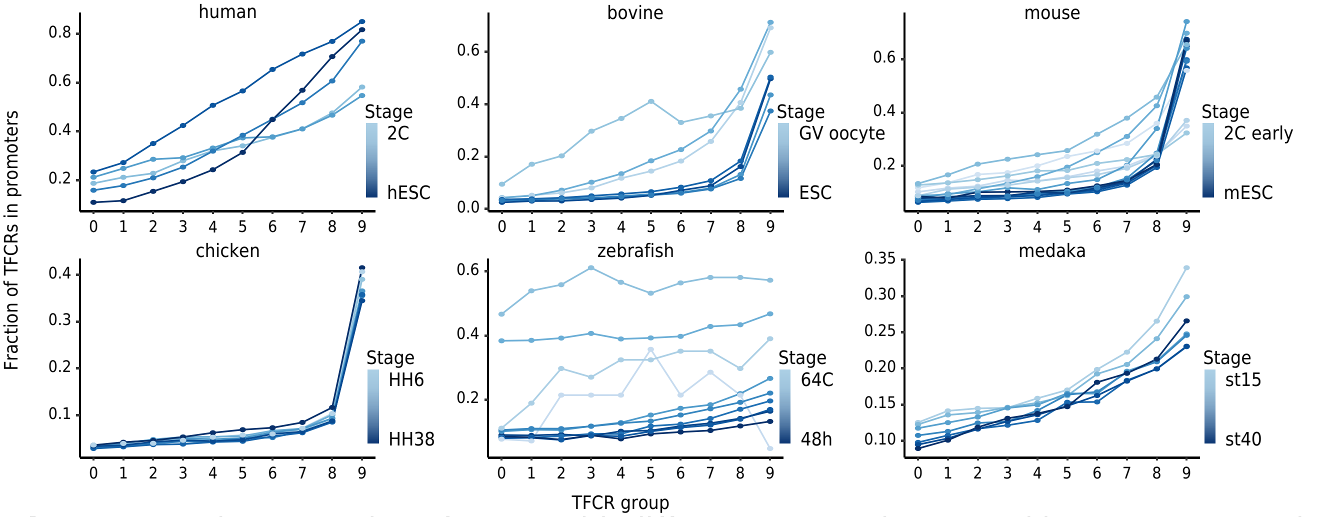

Figure S7. The proportion of TFCRs with different complexity located in promoters. Each line represents a stage. The color from light to deep represents the development of embryo stages.

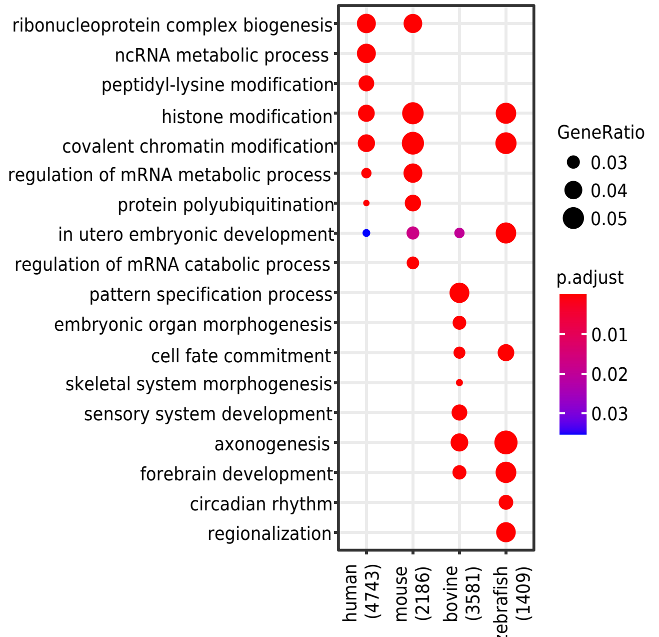

Figure S8. Gene ontology enrichment analysis of genes related with ZGA-gained TFCRs in human, mouse, bovine and zebrafish.

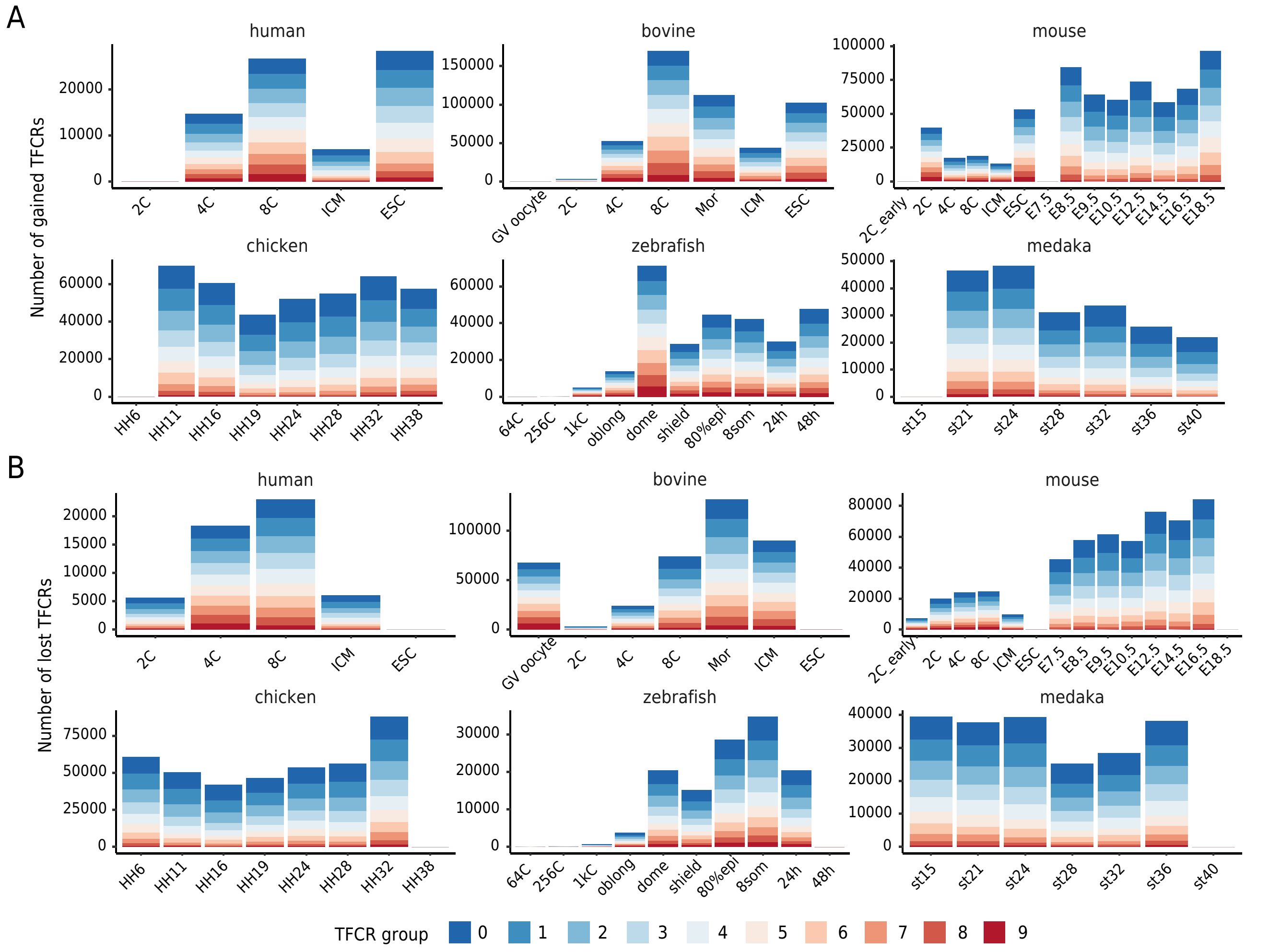

Figure S9. Number of dynamic TFCRs during embryo development. (A) The number of gained TFCRs in each stage compared to the consecutive stages. (B) The number of lost TFCRs in each stage. Gained TFCRs were compared to the previous stage. Lost TFCRs were compared to the next stage. The stacked color bars represent TFCRs with different complexities. Blue means the complexity lower than the median, and red means the complexity higher than the median. The color from blue to red means a gradual increase of TFCR complexity.

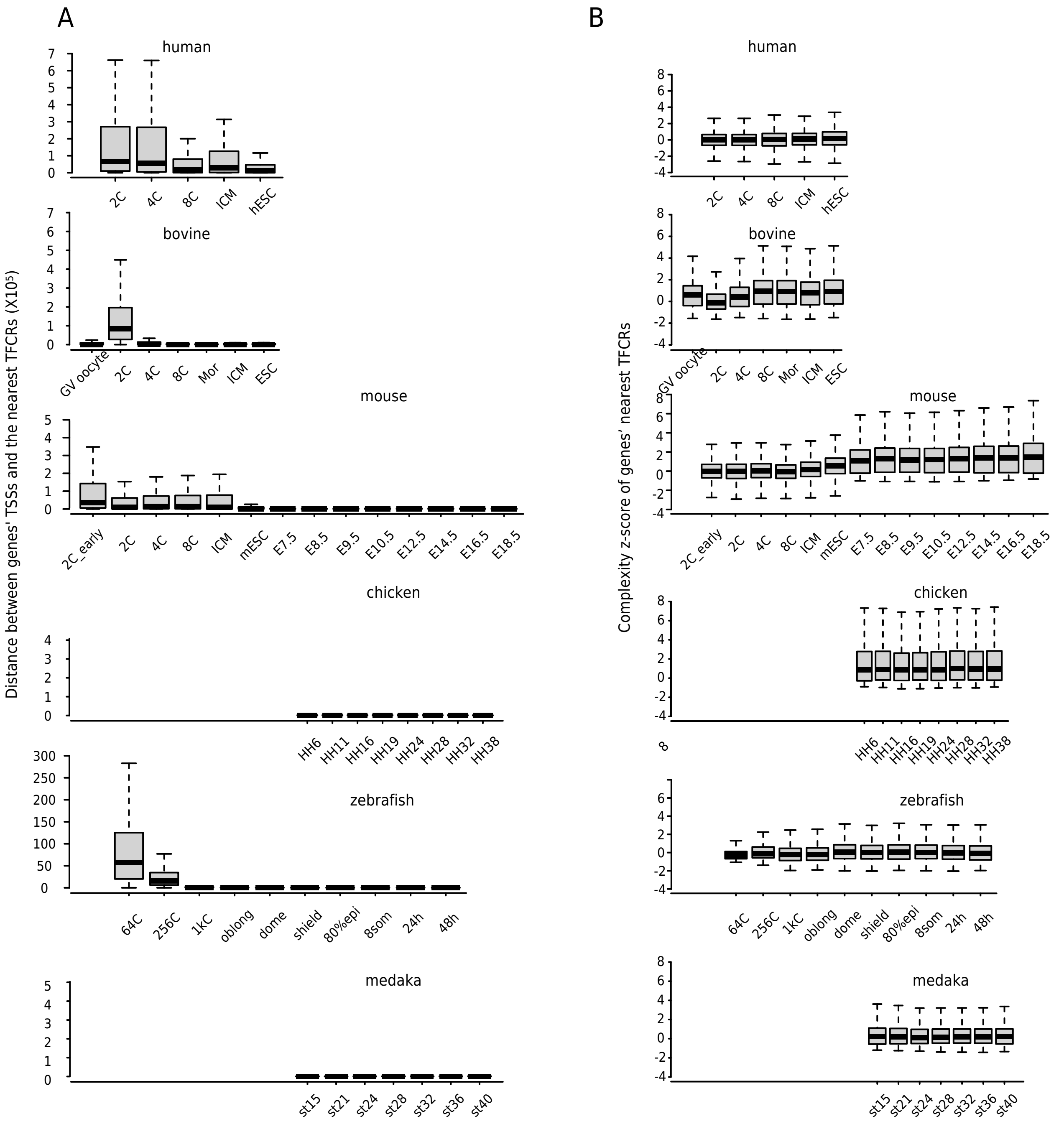

Figure S10. Nearest TFCRs to transcription start sites of genes among species.

(A) The distance of nearest TFCRs to transcription start sites of genes. (B) The complexity score of genes' nearest TFCRs. The complexity score was z-score scaled for each stage.

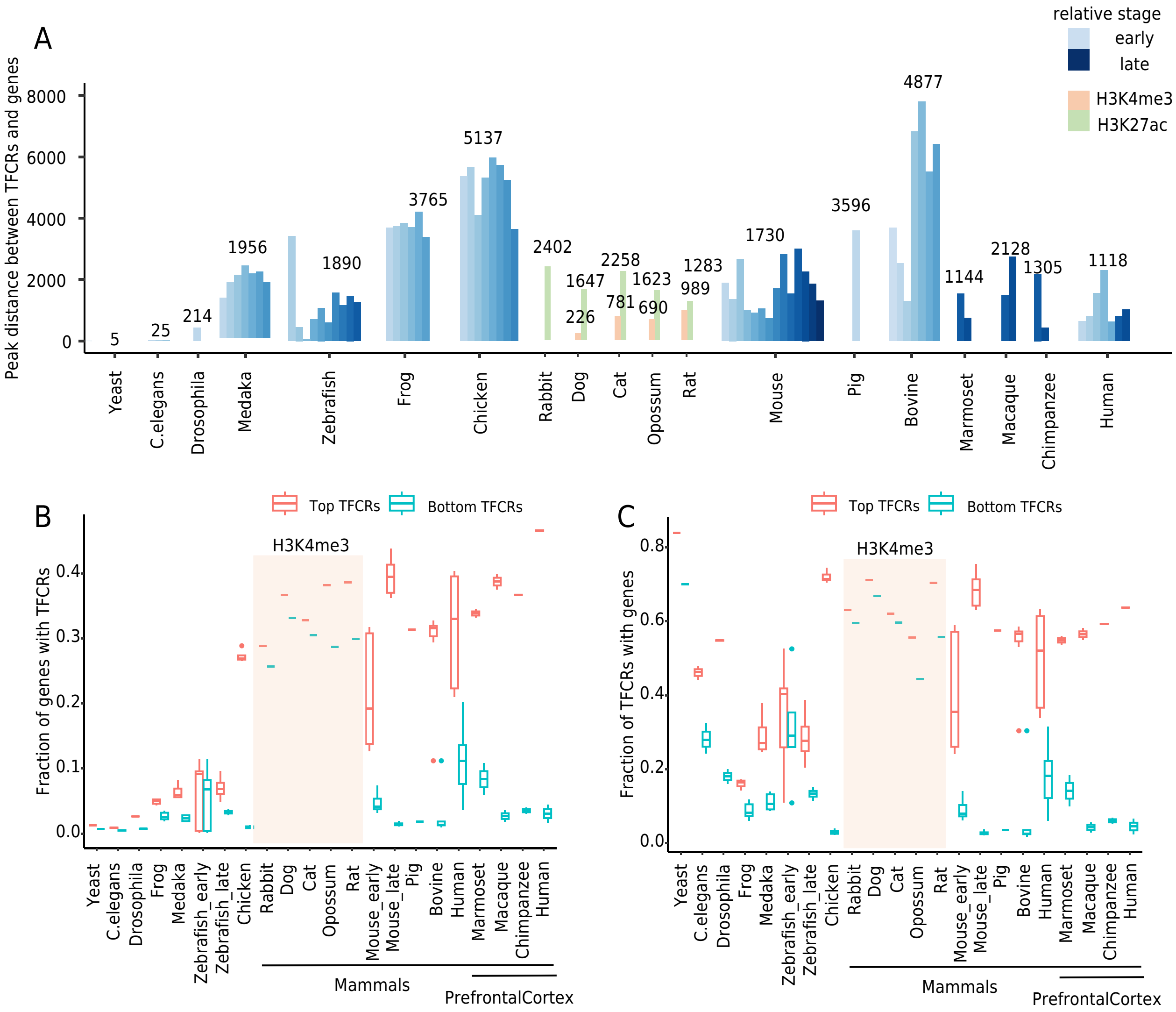

Figure S11. The characteristic of TFCRs from yeast to mammals. (A) The peak distance between TFCRs and its nearest genes among different species. The color from light to dark represents the development of embryo stages. The peak distance refers to the peak value of the density distribution of the distance between the TCFRs and promoters. (B) Fraction of genes associated with TFCRs. Top and bottom TFCRs are based on the 10% and 90% quantiles of TFCRs complexity. The TFCRs of rabbit, cat, dog, opossum, and rat were identified from ChIP-seq data of H3K4me3, and the other TFCRs were identified from ATAC-seq. (C) Fraction of TFCRs associated with genes.

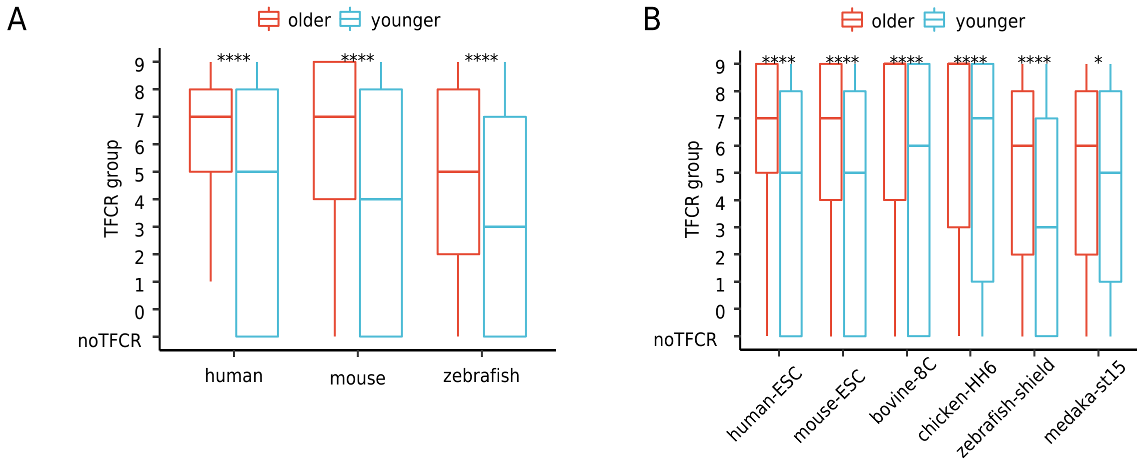

Figure S12. The complexity group of TFCRs in young and old genes among species.

(A) The complexity group of TFCRs in young and old genes at human ESC, mouse ESC and zebrafish shield. (B) The complexity group of TFCRs in young and old genes among six species. Categories of gene ages for other species were obtained based on their homologs to human. Statistical significance is evaluated using Wilcoxon test, ****p <= 0.0001, *p ≤ 0.05.

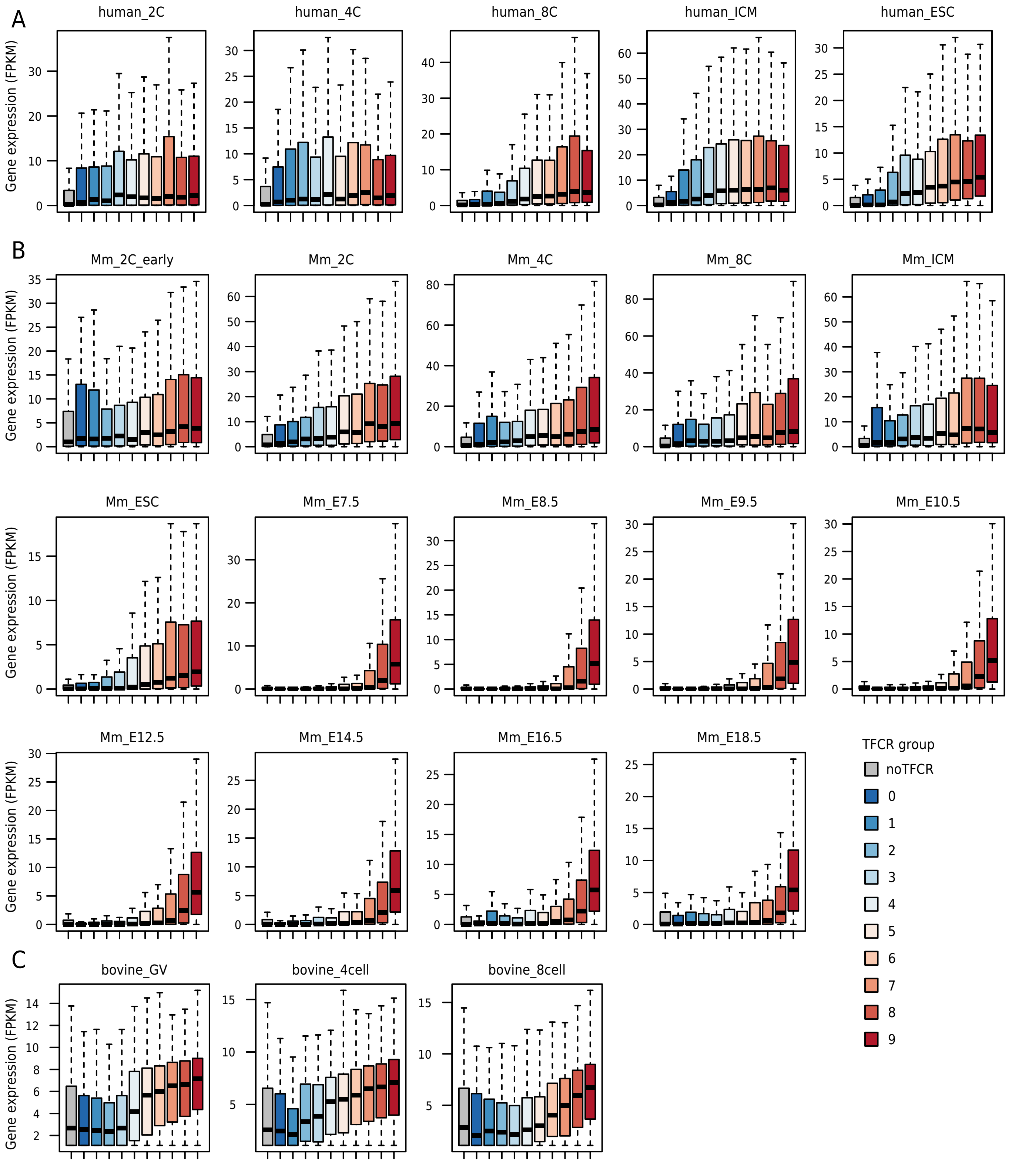

Figure S13. Expression level of genes associated with different complexity TFCRs. (Continued on next page, legend follows). Expression of genes at different stages in human (A), mouse (B), bovine (C), chicken (D), zebrafish (E), medaka (F).The grey color means that there is no TFCR located in gene's promoter. The color from blue to red means a gradual increase of TFCR complexity.

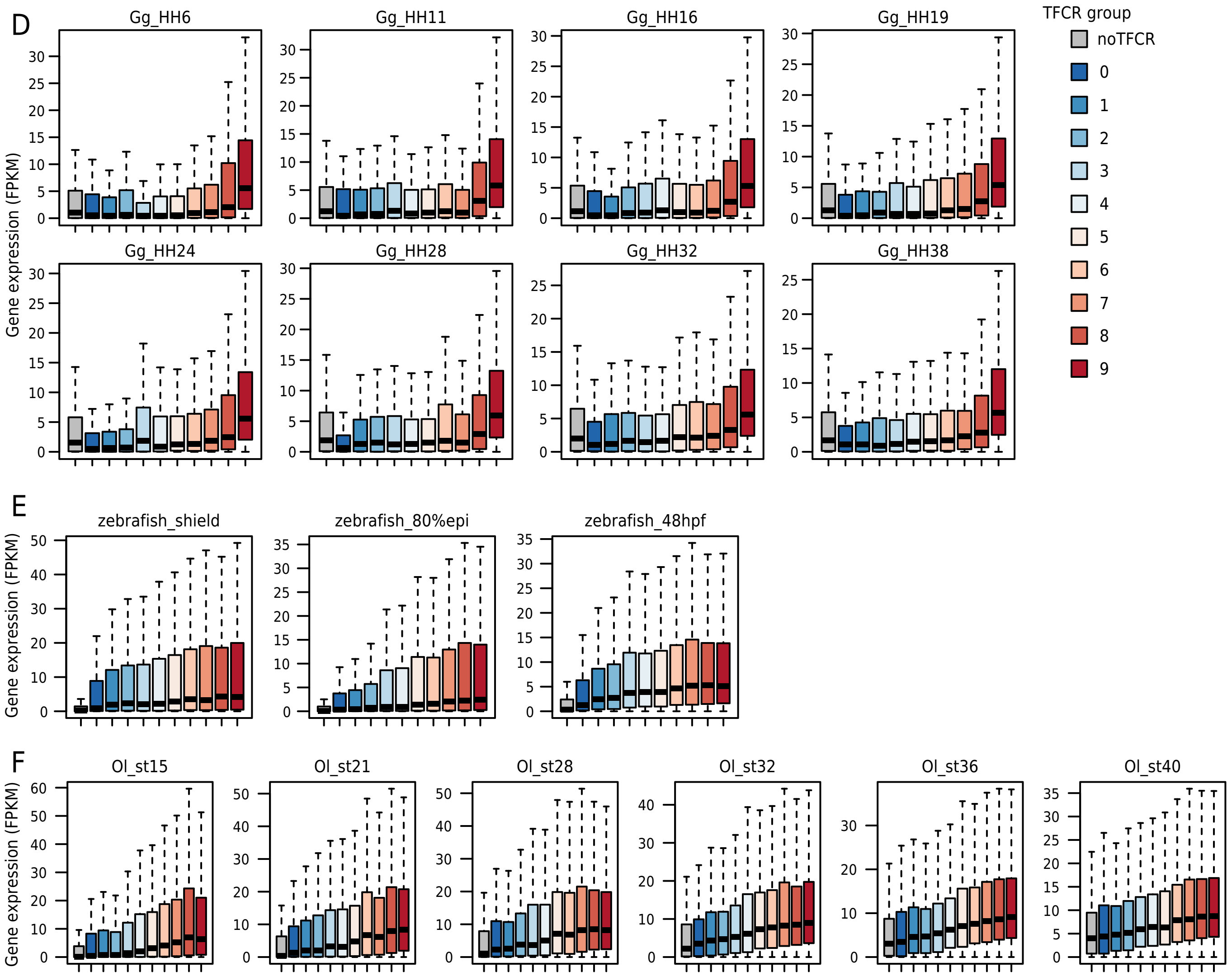

Figure S13. Expression level of genes associated with different complexity TFCRs. Expression of genes in human (A), mouse (B), bovine (C), chicken (D), zebrafish (E), medaka (F).The grey color means that there is no TFCR located in gene's promoter. The color from blue to red means a gradual increase of TFCR complexity.

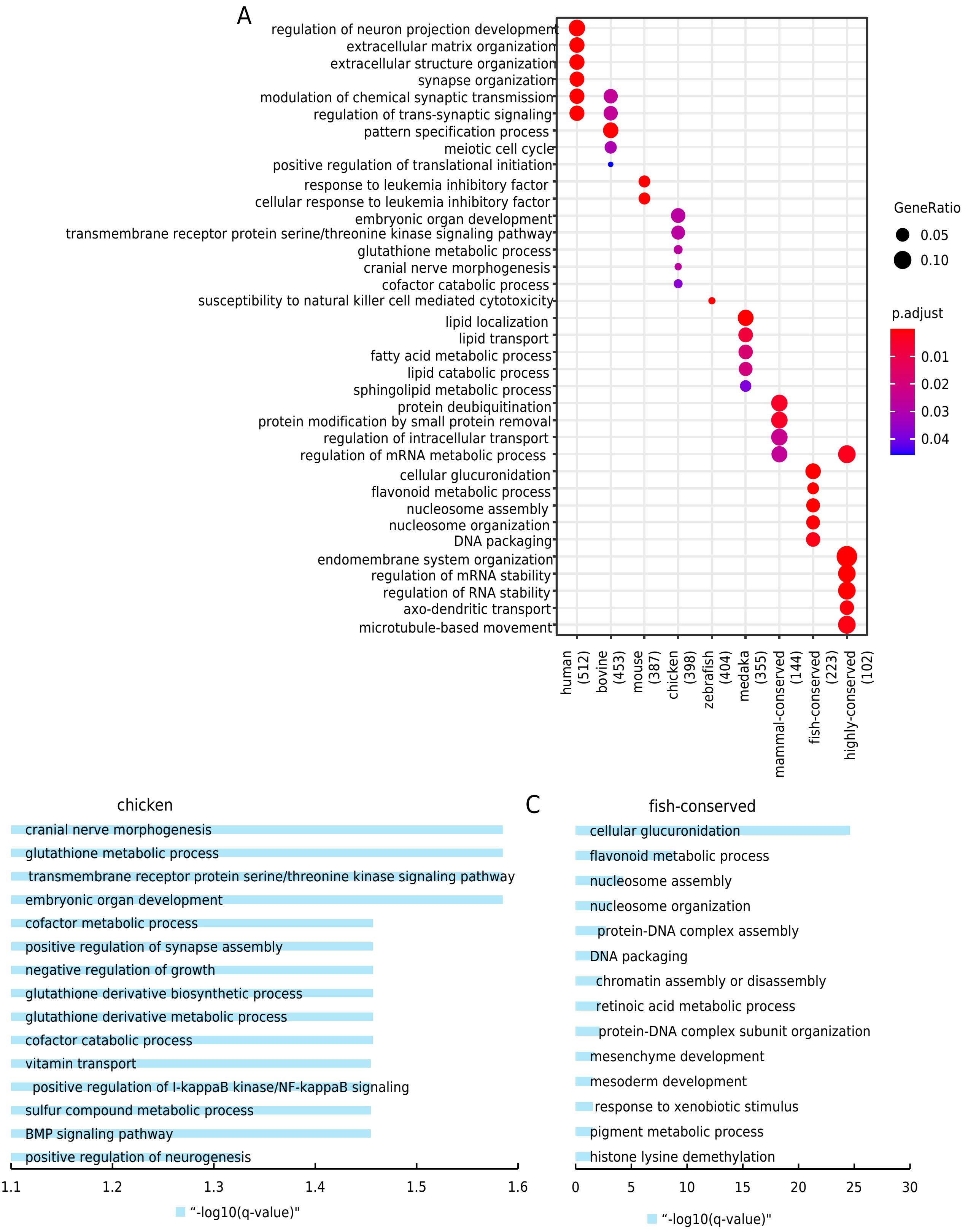

Figure S14. The characterization of genes with high RegulatoryScore among species.

(A) GO biological process of stage-specific genes with high RegulatoryScore during mouse embryo development. (B) GO biological process of chicken-specific genes with high RegulatoryScore. (C) GO biological process of fish-conserved genes with high RegulatoryScore. GO analysis is performed by clusterProfiler package with significance thresholds at p-value < 0.05 and q-value < 0.2.

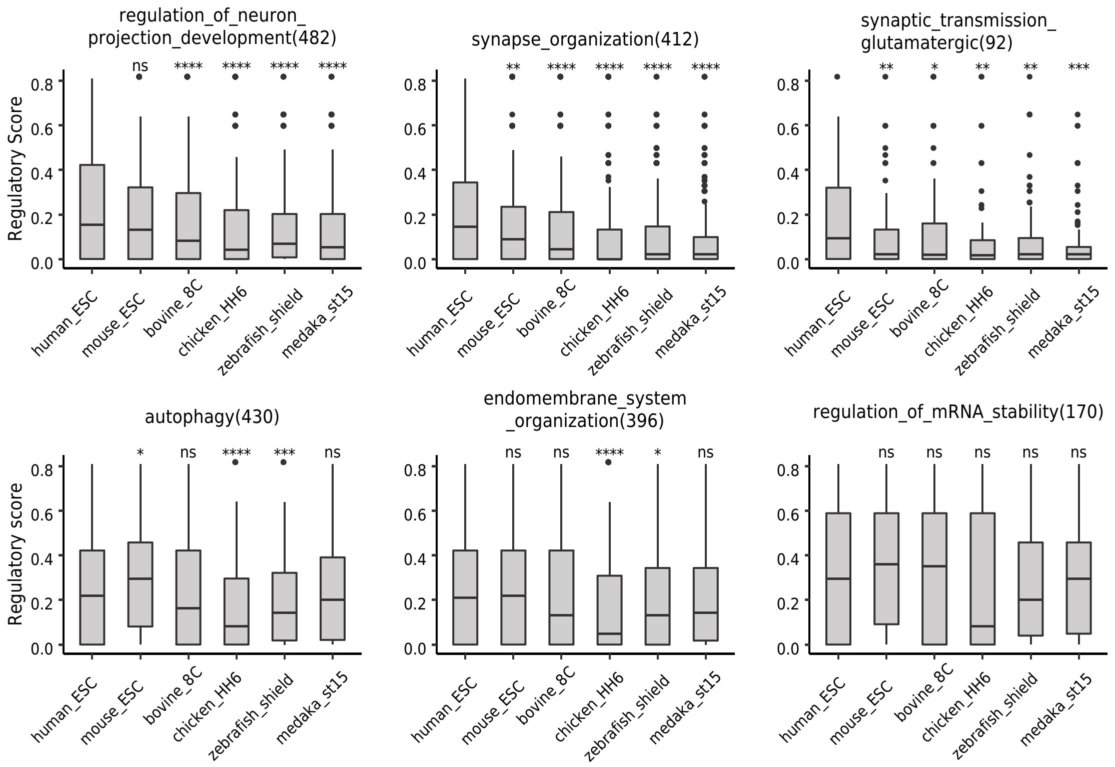

Figure S15. The RegulatoryScore of different gene sets among species. Gene sets are related to neuron development, synapse organization and other basic biological process. Statistical significance is evaluated using Wilcoxon test, ****p <= 0.0001, ***p <= 0.001, **p <= 0.01, *p ≤ 0.05, ns p > 0.05. The RegulatoryScore at human hESC was used as reference group, and each of other species was compared to the reference group.

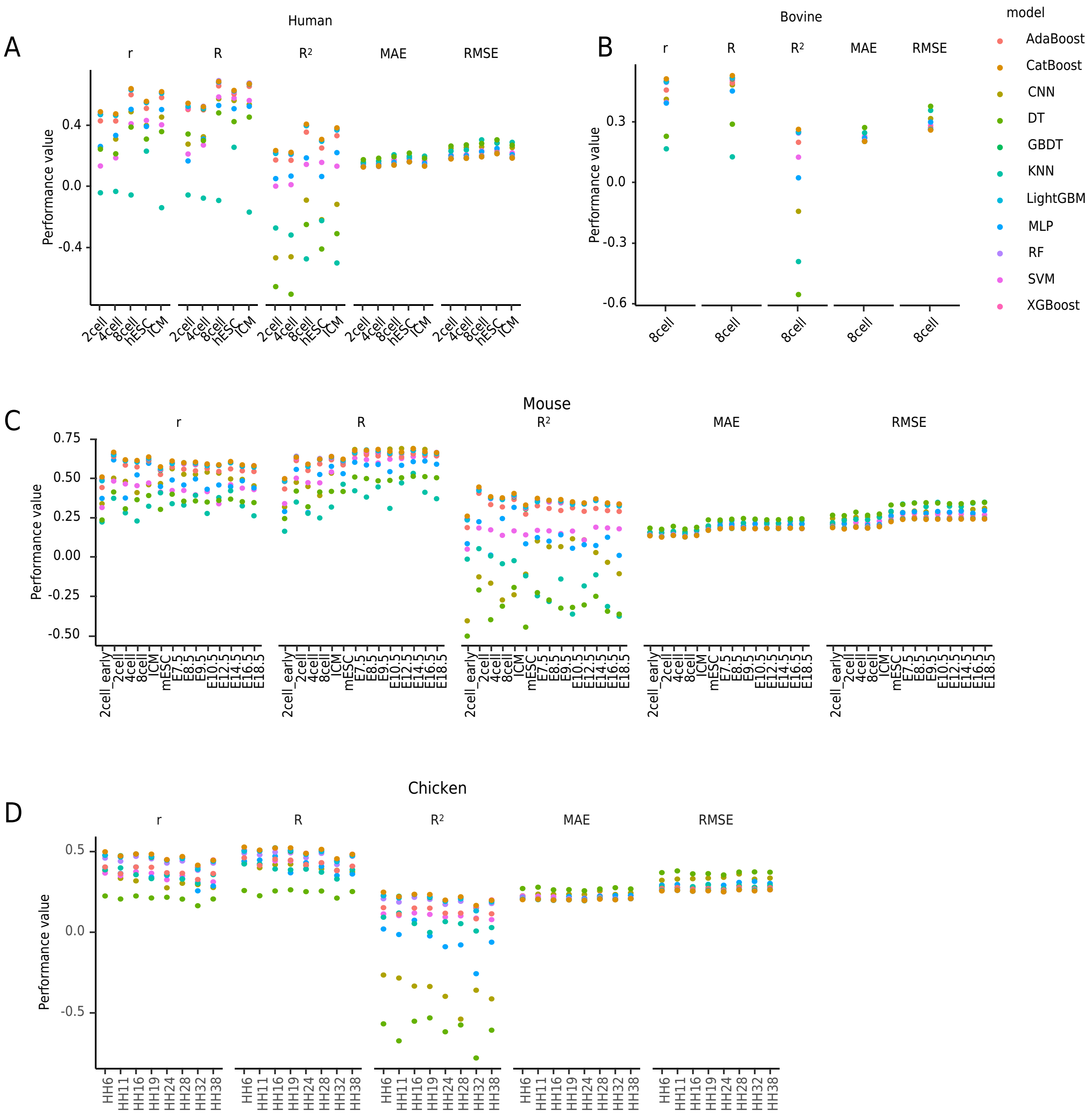

Figure S16. The performance values for eleven machine learning-based methods to predict the RegulatoryScore in different species (Continued on next page, legend follows). The performance values for eleven machine learning-based methods to predict the RegulatoryScore in human (A), bovine (B), mouse (C), chicken (D), medaka (E), zebrafish (F).

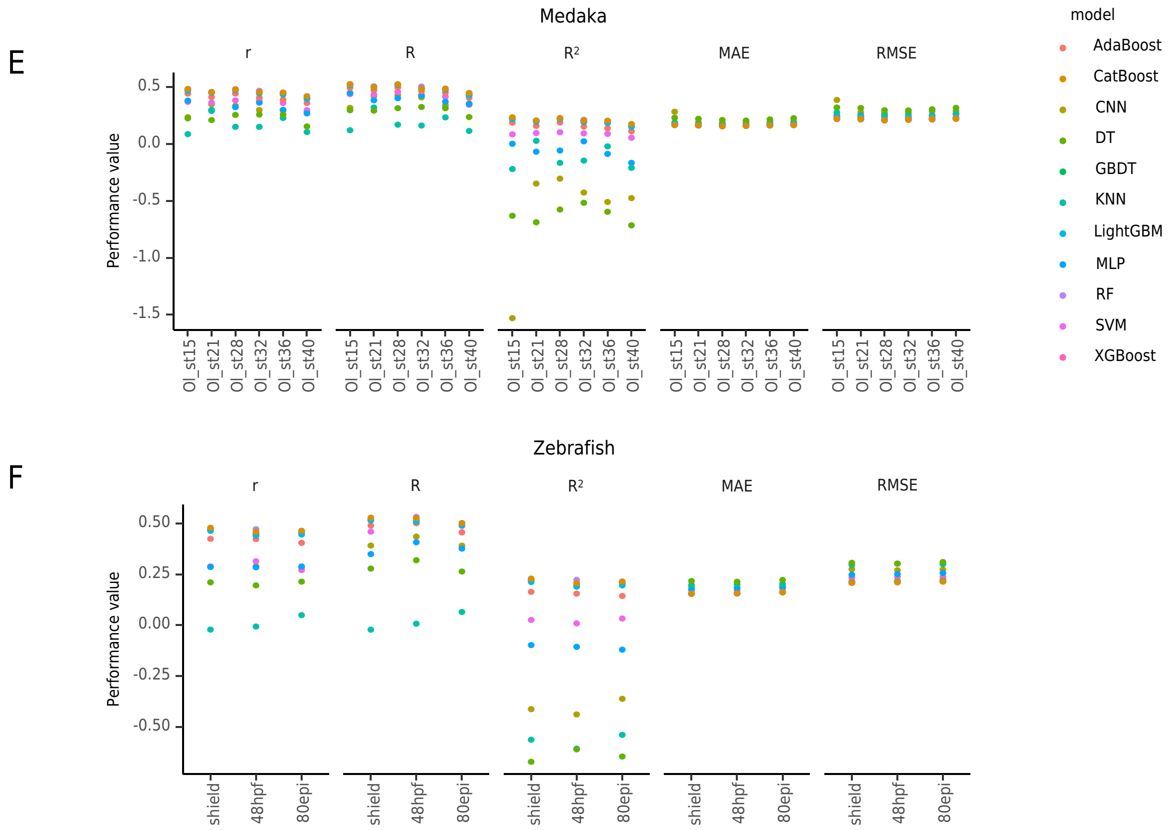

Figure S16. The performance values for eleven machine learning-based methods to predict the RegulatoryScore in different species. The performance values for eleven machine learning-based methods to predict the RegulatoryScore in human (A), bovine (B), mouse (C), chicken (D), medaka (E), zebrafish (F).

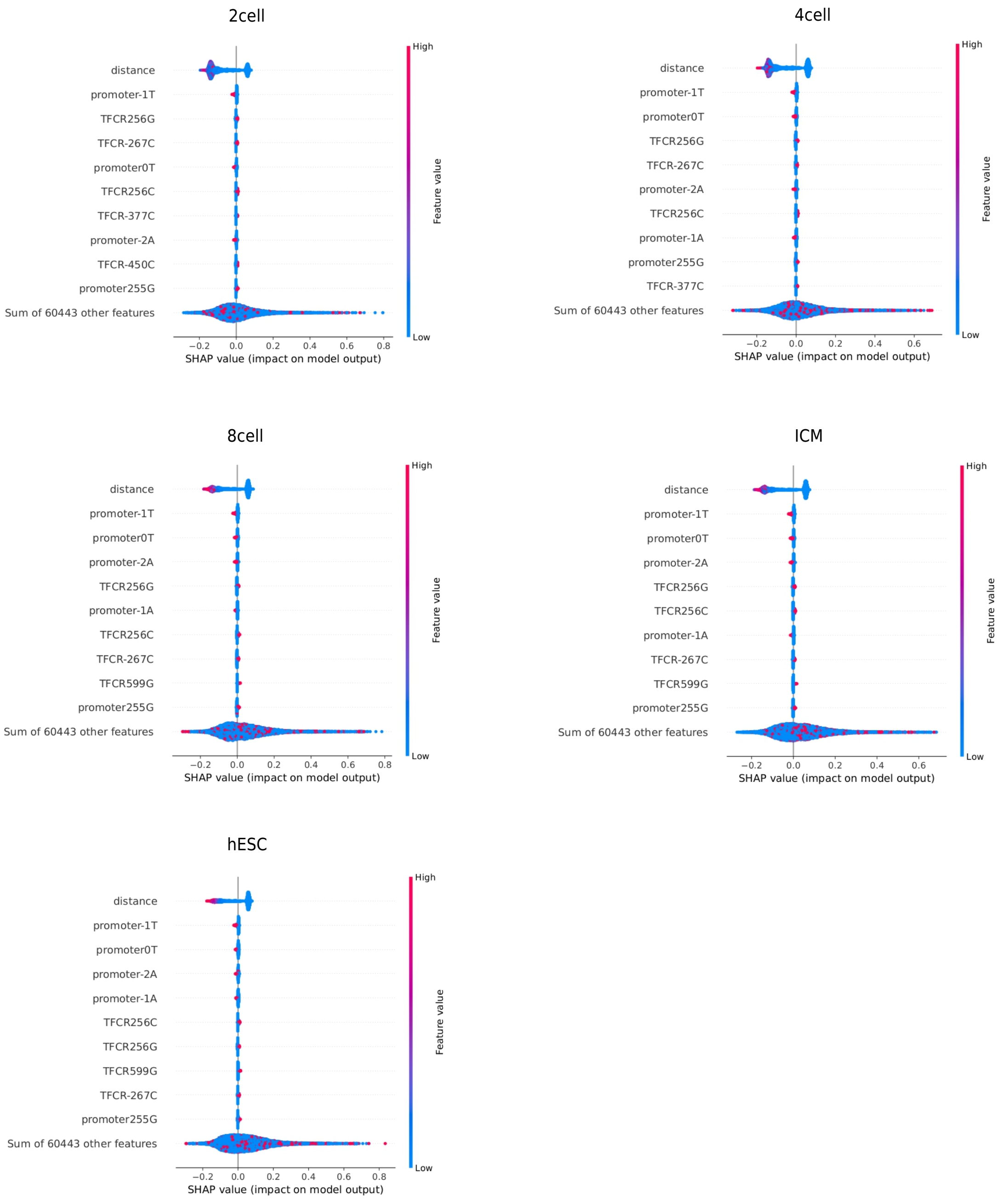

Figure S17. The distribution of SHAP value for top features in human. Red represents 1, blue represents 0. Red dots on the left indicate that the corresponding feature value is negatively correlated with the shap value, and the smaller the feature value, the higher the RS.

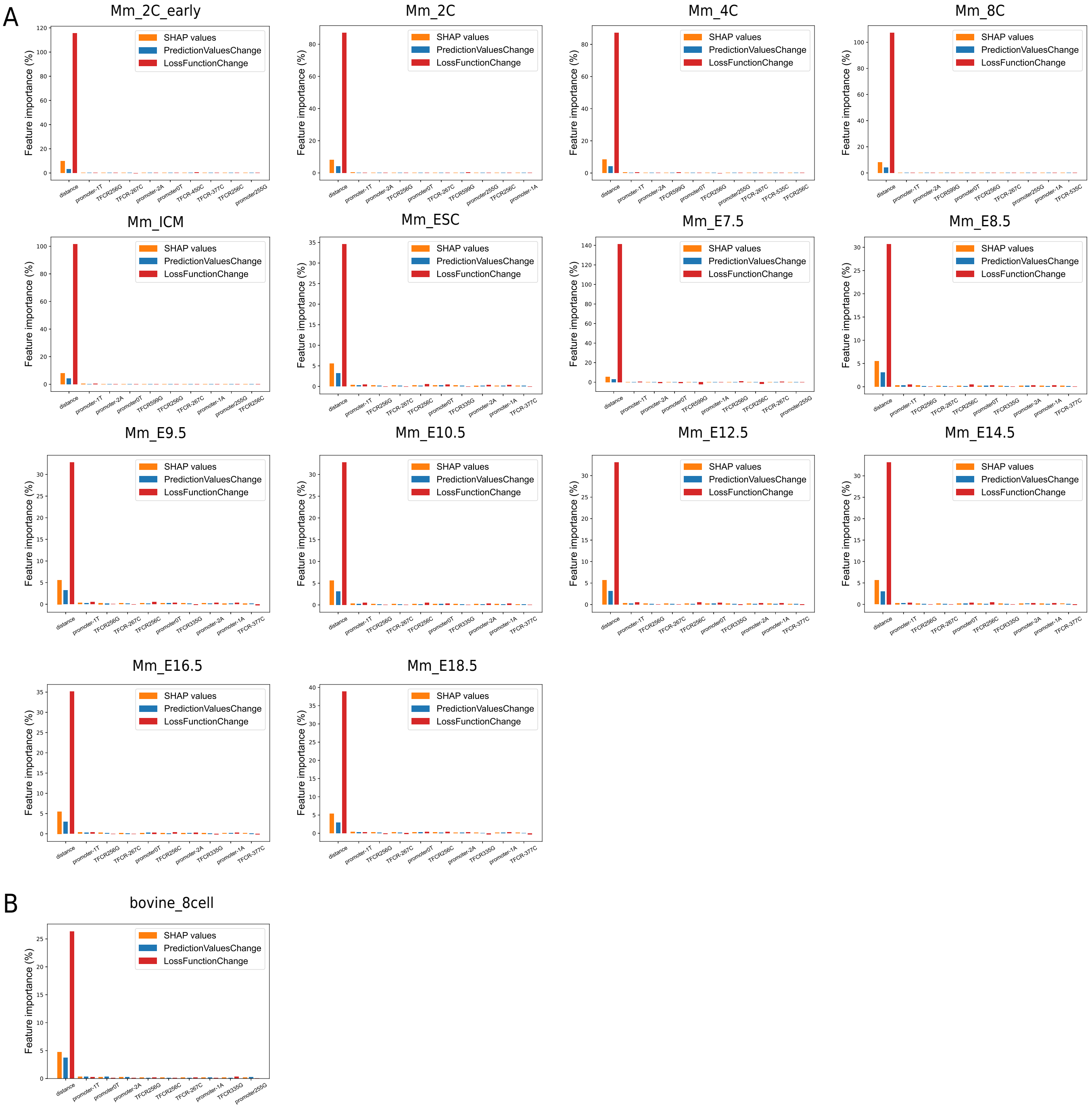

Figure S18. The SHAP values, PredicionValuesChange, LossFunctionChange for the feature importance in different species (Continued on next page, legend follows). The SHAP value, PredicionValuesChange, LossFunctionChange for the feature importance in mouse (A), bovine (B), chicken (C), zebrafish (D), medaka (E).

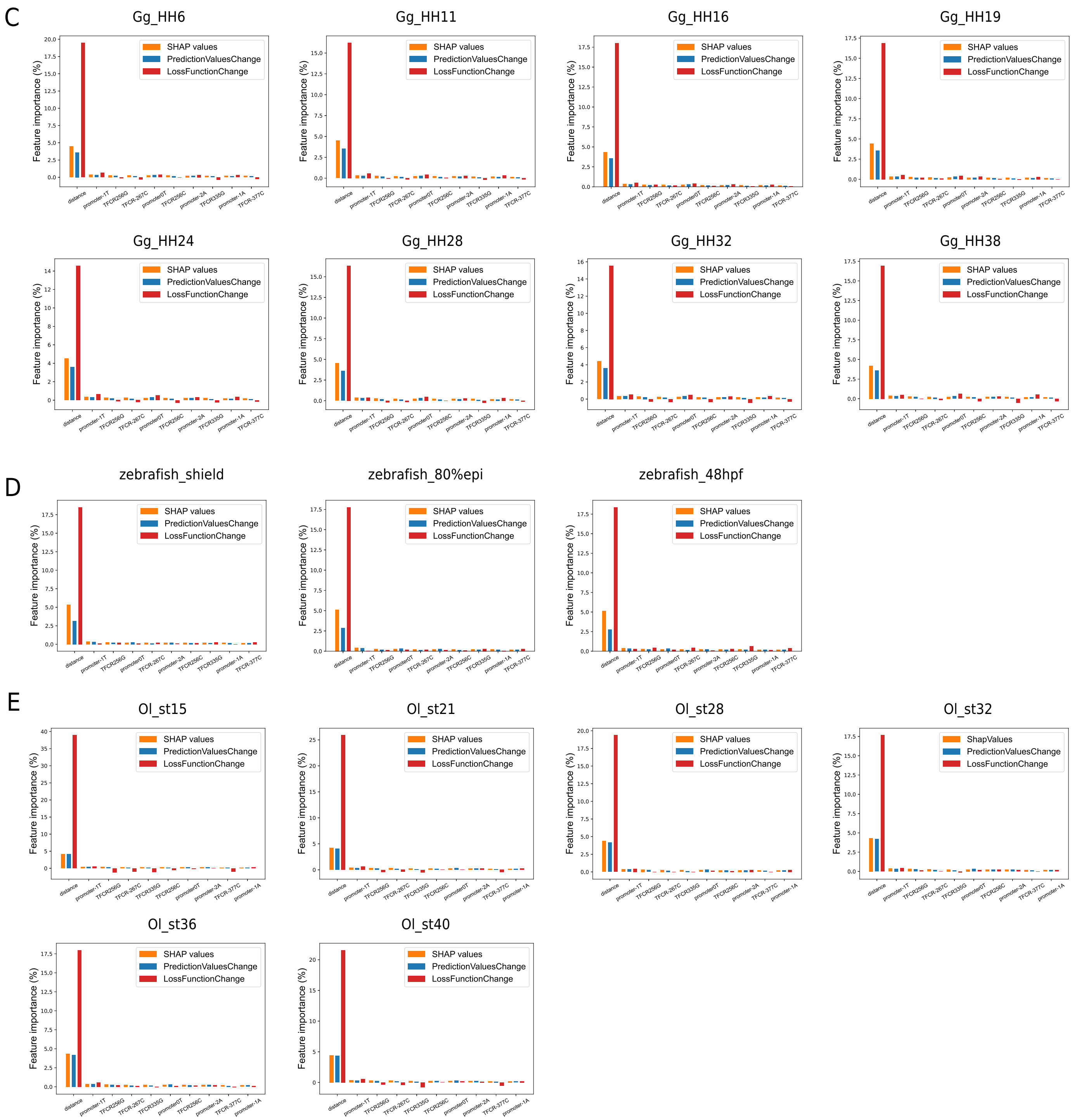

Figure S18. The SHAP values, PredicionValuesChange, LossFunctionChange for the feature importance in different species. The SHAP values, PredicionValuesChange, LossFunctionChange for the feature importance in mouse (A), bovine (B), chicken (C), zebrafish (D), medaka (E).

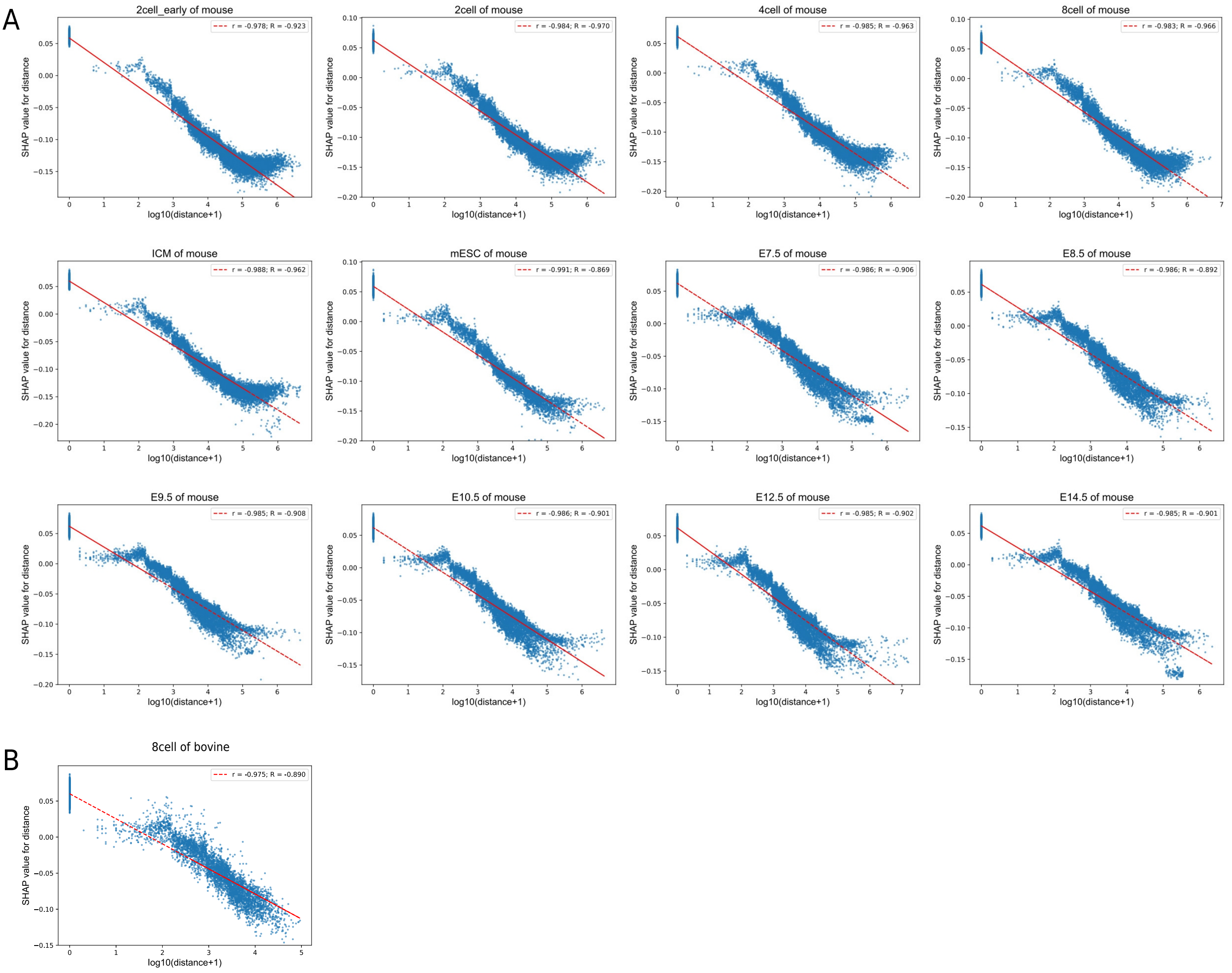

Figure S19. The relationship between SHAP values of distance and distances for each stage in different species (Continued on next page, legend follows). The relationship between SHAP values of distance and distances for each stage in mouse (A), bovine (B), chicken (C), zebrafish (D), medaka (E). Pearson correlation coefficient was represented as r. Spearman correlation coefficient was represented as R.

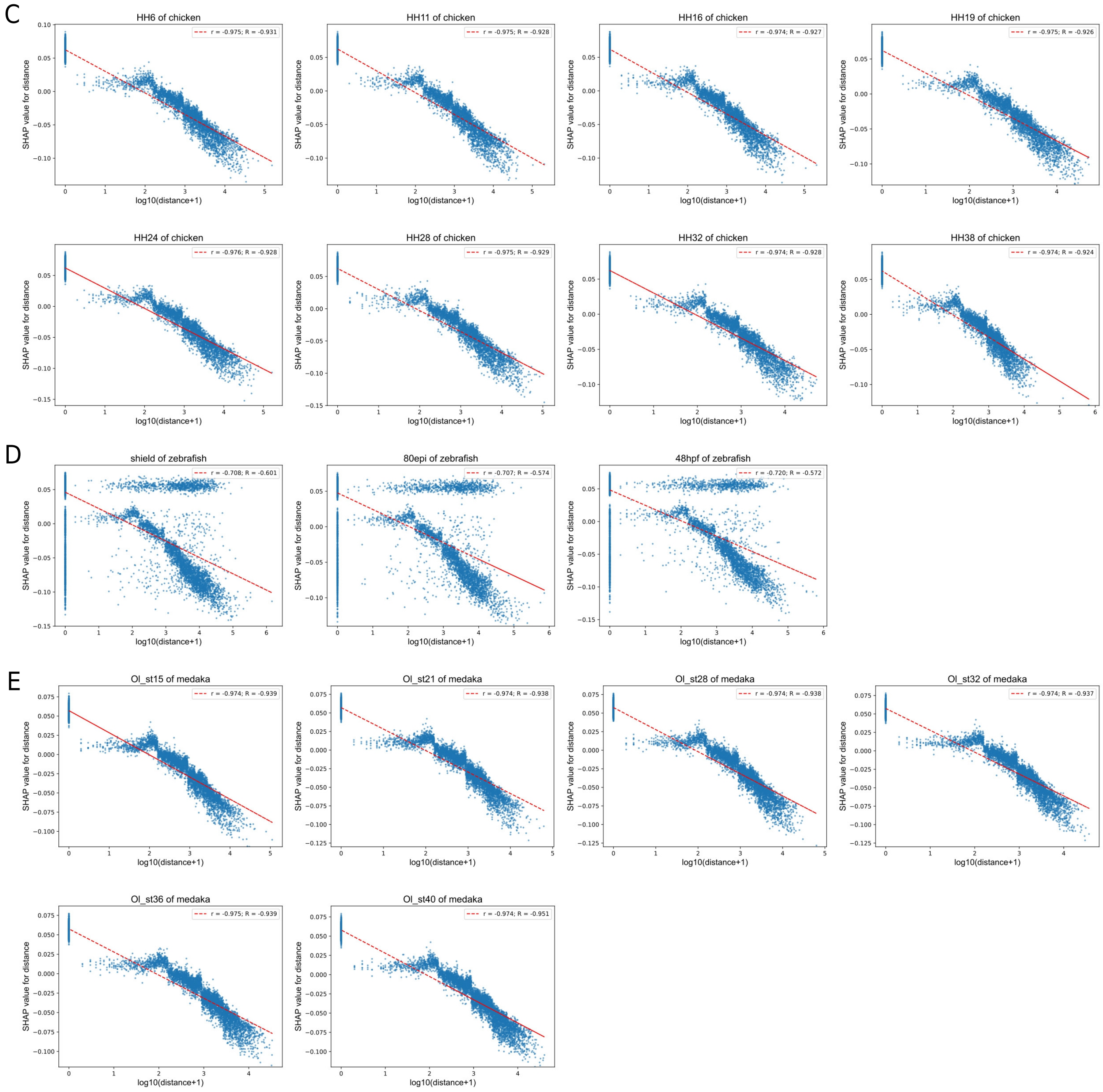

Figure S19. The relationship between SHAP values of distance and distances for each stage in different species. Pearson correlation coefficient was represented as r. Spearman correlation coefficient was represented as R. The relationship between SHAP values of distance and distances for each stage in mouse (A), bovine (B), chicken (C), zebrafish (D), medaka (E).

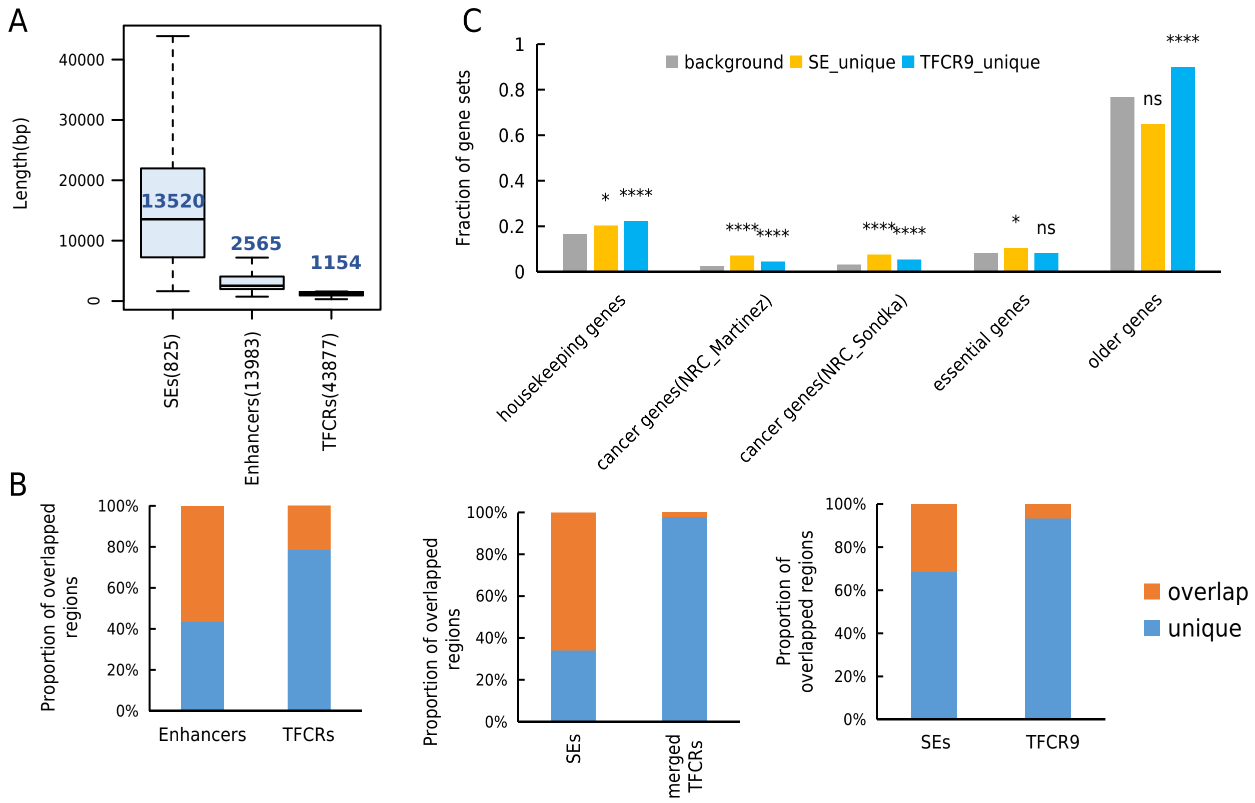

Figure S20. The comparison between TFCRs and super-enhancers. (A) The length of SEs, enhancers and TFCRs. The median lengths of elements were labeled on the plots. The numbers of SEs, enhancers and TFCRs were shown in parentheses. (B) The proportion of overlapped regions between enhancers and TFCRs, SEs and merged TFCRs, SEs and TFCR9. (C) The enrichment of genes associated with SEs and TFCR9 in different gene sets. Grey bars represent background and show the fraction of different gene sets in all protein-coding genes. Statistical significance is given by hypergeometric test. ****p <= 0.0001, ***p <= 0.001, **p <= 0.01, *p ≤ 0.05, ns p > 0.05.
