# Supplementary material for: The developmental and evolutionary characteristics of transcription factor binding site clustered regions based on an explainable machine learning model
Source: Nucleic Acids Res. 2024 May 30;52(13):7610–26. doi: 10.1093/nar/gkae441 (PMC11260490; doi:10.1093/nar/gkae441)
Supplement: gkae441_Supplemental_Files [file gkae441_supplemental_files.zip › Supplementary Methods_revised.docx]

## Supplementary Methods

### Peak calling and the comparability of peak distribution among species

All peaks are identified by MACS2 ^1^ but with some different parameters such as shift, extsize and q-value or p-value. We tested these parameters described in the previous papers (**Table S1**) on human ESC ATAC-seq data and found that different parameters have an impact on the number of peaks identified (**Table S2**). However, when the q-value is unchanged, the changes of shift and extsize have almost no effect on the genomic distribution of peaks (**Figure S1A**). The peak calling parameters for human and mouse (2-cell early ~ mESC) were the same. The significant q-values for peak calling were the same in human, mouse and bovine. Therefore, the results between human, mouse and bovine were comparable. The sequence depth and peak calling parameters for mouse (E7.5~E18.5), medaka, and chicken were the same, so the results among them were also comparable. The similar genomic distribution between mouse (2-cell early ~ mESC) and mouse (E7.5~E18.5) showed the comparability of those two datasets (**Figure 1E**). Therefore, although there is a difference in parameters for peak calling in different species, these results are still comparable.

### Identification of TFCRs

First, the TFBSs were identified by FIMO ^2^. The position-specific weight matrices (PWMs) of transcription factors were downloaded from CIS-BP databases ^3^ (**Table S3**). Average numbers of identified TFBSs located within 5kb genomic bins were significantly higher compared to shuffled TFBSs among all species (Wilcoxon rank-sum test p-value < 0.0001), especially in human (**Table S4**). This result showed that identified TFBSs were more closely distributed than randomly shuffled TFBSs (**Figure S2**), indicating the clustering characteristic of TFBSs in those species. The genomic sequences under the open chromatin regions were used as inputs for FIMO with a custom library of all motifs for each species to scan for motif instances at a p-value threshold of 10^-5^. Then, an established method ^4, 5^ was used to identify TFCRs by performing the Gaussian kernel density estimations across the genome (with a bandwidth of 300bp centered on each TFBS). Each peak in density profile was considered a TFCR. To determine the complexity of each TFCR, the Gaussian kernelized distances from each peak that contributed at least 0.1 to its strength were determined.

### The calculation of TFCR complexity

The complexity of each TFCR was determined by the quantity and proximity of the contributing TFBS. We combined motif instances based on the TF family information from CIS-BP to calculate the complexity of TFCR. That means, if multiple motifs bunded the same sites, we only calculate the number of different TF families to which they belong as the complexity of TFCR. For example, Jun, FOS and AP1 all come from the bZIP family. When these three TFs appear in the same location, the complexity only considers the number of TF family they belong to, that is, 1. The window for each TFCR was determined by finding the maximum distance (in bp) from the TFCR to a contributing TF and then adding 150 bp (one-half of the bandwidth). Each window was centered on the TFCR. The identified TFCR was grouped into 10 groups based on their complexity from low to high.

We also identified TFCRs using motifs from JASPAR ^6^ for each species. About 98% of TFCRs identified using JASPAR were overlapped with those identified using CIS-BP (**Table S5**. Here, TFCRs that share over 50% base pairs are defined as overlapped). These results show that the identification of TFCRs using CIS-BP is robust.

### Robustness of TFCRs identification under different sequencing depth

Different sequencing depths may lead to bias when comparing TFCR. To assess the effect of sequencing depth on identification results, we downsampled ATAC-seq data from human embryonic stem cell samples, generating sequencing samples with 5 million reads to 40 million reads with a step size of 5 million reads. The results showed that the number of identified open chromatin regions and TFCRs increased with sequencing depth, but the magnitude of the increase gradually decreased and was stable at 35 million reads (**Figure S3A**). The distribution of open chromatin regions was similar to that of TFCR in genomic elements, and the proportion of chromatin in the promoter region decreased at first and then stabilized at 37% with the increase of sequencing depth (**Figure S3B**). When TFCR was divided into 10 groups according to its complexity from low to high, under the condition of different sequencing depth, the proportion of identified TFCRs located in the promoter increased with the increase of TFCRs’ complexity (**Figure S3C**).

Furthermore, we selected SOX2, an important gene in early embryonic development, to analyze the distribution of TFCRs surrounding it under different sequencing depths. SOX2 is a transcription factor essential for regulating mammalian embryonic development and maintaining the self-renewal and multipotency of embryonic stem cells. It was found that the TFCRs located in the promoter region of SOX2 can always be identified, even under different sequencing depths, and its complexity is consistent (**Figure S3D**). These results suggested that the identification of TFCRs was robust at different sequencing depths, while TFCRs located in the promoter region were more likely to be identified at lower sequencing depths.

### Robustness of RegulatoryScore under different sequencing depth

In order to evaluate the robustness of RegulatoryScore, we used downsampling ATAC-seq data of hESC to compare the correlation of RegulatoryScore under different sequencing depths. The results showed that when the sequencing depth was greater than 10 million reads, the Pearson correlations of RegulatoryScore among different sequencing depths were high (from 0.62 to 0.97) (**Figure S4**), which indicated that RegulatoryScore was less affected by the sequencing depth and was a relatively robust index.

## Supplementary figure legends

**Figure S1. The genomic distribution of ATAC-seq peaks.**

(A) The genomic distribution of ATAC-seq peaks at hESC called by MACS2 using different parameters. (B) The genomic distribution of ATAC-seq peaks among species.

**Figure S2. The density plot of the number of TFBSs located in 5kb genomic bins within each species.**

Red and blue lines represent the distribution of the number of identified and randomly shuffled TFBSs within 5kb genomic bins, respectively. x axis is log10 scaled.

**Figure S3. The robustness of TFCR identification under different sequencing depths.**

(A) The number of ATAC-seq peaks and TFCRs identified from different sequencing depths. (B) The distribution of ATAC-seq peaks and TFCRs in functional elements in gemome at different sequencing depths. (C) The fraction of TFCRs located in promoters at different sequencing depths. (D) The overview of TFCRs surrounding SOX2 at different sequencing depths. M: million.

**Figure S4. The Pearson correlation of RegulatoryScore calculated from different sequencing depths.**

**Figure S5. Genome coverage of TFCRs across species.**

(A) Genome coverage of idengaintified TFCRs across species. (B) Genome coverage of TFCRs identified from subsampled 30 million ATAC-seq uniquely mapped reads from human, mouse and bovine. (C) The number of TFCRs identified from subsampled ATAC-seq peaks. ATAC-seq peak sets were called from subsampled 30 million ATAC-seq uniquely mapped reads from human,mouse and bovine. (D) The genomic distribution of TFCRs identified from subsampled ATAC-seq peak sets of human, mouse and bovine.

**Figure S6. The fraction of ATAC-seq peaks and TFCRs in promoters.**

(A) The proportion of identified TFCRs and randomly shuffled TFCRs in the promoter. We shuffled the identified TFCRs in each species by randomly switching the positions of TFCRs in the genome and compared the location of shuffled TFCRs with identified TFCRs. (B) The fraction of ATAC-seq peaks and TFCRs in promoters.

**Figure S7. The proportion of TFCRs with different complexity located in promoters.**

Each line represents a stage. The color from light to dark represents the development of embryo stages.

**Figure S8. Gene ontology enrichment analysis of genes related with ZGA-gained TFCRs in human, mouse, bovine and zebrafish.** In order to compare the enriched GO terms between species, we converted the ZGA-gained genes of mouse, bovine and zebrafish into the homology gene of human and then annotated it with GO terms from human.

**Figure S9. Number of dynamic TFCRs during embryo development.**

(A) The number of gained TFCRs in each stage compared to the consecutive stages. (B) The number of lost TFCRs in each stage. Gained TFCRs were compared to the previous stage. Lost TFCRs were compared to the next stage. The stacked color bars represent TFCRs with different complexities. Blue means the complexity lower than the median, and red means the complexity higher than the median. The color from blue to red means a gradual increase of TFCR complexity.

**Figure S10. Nearest TFCRs to transcription start sites of genes among species.**

(A) The distance of nearest TFCRs to transcription start sites of genes. (B) The complexity score of genes’ nearest TFCRs. The complexity score was z-score scaled for each stage.

**Figure S11. The characteristic of TFCRs from yeast to mammals.** (A) The peak distance between TFCRs and its nearest genes among different species. The color from light to dark represents the development of embryo stages. The peak distance refers to the peak value of the density distribution of the distance between the TCFRs and promoters. (B) Fraction of genes associated with TFCRs. Top and bottom TFCRs are based on the 10% and 90% quantiles of TFCRs complexity. The TFCRs of rabbit, cat, dog, opossum, and rat were identified from ChIP-seq data of H3K4me3, and the other TFCRs were identified from ATAC-seq. (C) Fraction of TFCRs associated with genes.

**Figure S12. The complexity group of TFCRs in young and old genes among species.**

(A) The complexity group of TFCRs in young and old genes at human ESC, mouse ESC and zebrafish shield. (B) The complexity group of TFCRs in young and old genes among six species. Categories of gene ages for other species were obtained based on their homologs to human. Statistical significance is evaluated using Wilcoxon test, ****p <= 0.0001, *p ≤ 0.05.

**Figure S13. Expression level of genes associated with different complexity TFCRs.**

Expression of genes at different stages in human (A), mouse (B), bovine (C), chicken (D), zebrafish (E), medaka (F). The grey color means that there is no TFCR located in gene’ promoter. The color from blue to red means a gradual increase of TFCR complexity.

**Figure S14. The characterization of genes with high RegulatoryScore among species.**

(A) GO biological process of stage-specific genes with high RegulatoryScore during mouse embryo development. (B) GO biological process of chicken-specific genes with high RegulatoryScore. (C) GO biological process of fish-conserved genes with high RegulatoryScore. GO analysis is performed by clusterProfiler package with significance thresholds at p-value < 0.05 and q-value < 0.2.

**Figure S15. The RegulatoryScore of different gene sets among species.**

Gene sets are related to neuron development, synapse organization and other basic biological process. Statistical significance is evaluated using Wilcoxon test, ****p <= 0.0001, ***p <= 0.001, **p <= 0.01, *p ≤ 0.05, ns p > 0.05. The RegulatoryScore at human hESC was used as reference group, and each of other species was compared to the reference group.

**Figure S16.** **The performance values for eleven machine learning-based methods to predict the RegulatoryScore in different species.** The performance values for eleven machine learning-based methods to predict the RegulatoryScore in human (A), bovine (B), mouse (C), chicken (D), medaka (E), zebrafish (F).

**Figure S17. The distribution of SHAP value for top features in human.** Red represents 1, blue represents 0. Red dots on the left indicate that the corresponding feature value is negatively correlated with the shap value, and the smaller the feature value, the higher the RS.

**Figure S18. The SHAP values, PredicionValuesChange, LossFunctionChange for the feature** **importance in different species.** The SHAP values, PredicionValuesChange, LossFunctionChange for the feature importance in mouse (A), bovine (B), chicken (C), zebrafish (D), medaka (E).

**Figure S19.** **The relationship between SHAP values of distance and distances for each stage in different species.** The relationship between SHAP values of distance and distances for each stage in mouse (A), bovine (B), chicken (C), zebrafish (D), medaka (E). Pearson correlation coefficient was represented as r. Spearman correlation coefficient was represented as R.

**Figure S20.** **The comparison between TFCRs and super-enhancers.** (A) The length of SEs, enhancers and TFCRs. The median lengths of elements were labeled on the plots. The numbers of SEs, enhancers and TFCRs were shown in parentheses. (B) The proportion of overlapped regions between enhancers and TFCRs, SEs and merged TFCRs, SEs and TFCR9. (C) The enrichment of genes associated with SEs and TFCR9 in different gene sets. Grey bars represent background and show the fraction of different gene sets in all protein-coding genes. Statistical significance is given by hypergeometric test. ****p <= 0.0001, ***p <= 0.001, **p <= 0.01, *p ≤ 0.05, ns p > 0.05.

## Supplementary files. Feature analysis charts for the distance feature. Feature analysis charts provide a calculated and plotted set of statistics for the distance feature. The X-axis of the chart contains values of the distance divided into buckets. The Y-axis contains the graphs of average target value (RegulatoryScore) in the bucket (bule line), average predictied RegulatoryScore in the bucket (red dotted line), number of samples in the bucket (green box) and average predictied RegulatoryScore on varying values of the distance (purple dotted line). To calculate the last graph, the value of the distance is successively changed to fall into every bucket for every input sample. The value for a bucket on the graph is calculated as the average for all samples when their distance values are changed to fall into this bucket.

**Reference**

1 Feng J, Liu T, Qin B, Zhang Y, Liu XS. Identifying ChIP-seq enrichment using MACS. *Nat Protoc* 2012; **7**:1728-1740.

2 Grant CE, Bailey TL, Noble WS. FIMO: scanning for occurrences of a given motif. *Bioinformatics* 2011; **27**:1017-1018.

3 Weirauch MT, Yang A, Albu M *et al.* Determination and inference of eukaryotic transcription factor sequence specificity. *Cell* 2014; **158**:1431-1443.

4 mod EC, Roy S, Ernst J *et al.* Identification of functional elements and regulatory circuits by Drosophila modENCODE. *Science* 2010; **330**:1787-1797.

5 Chen H, Li H, Liu F *et al.* An integrative analysis of TFBS-clustered regions reveals new transcriptional regulation models on the accessible chromatin landscape. *Sci Rep* 2015; **5**:8465.

6 Castro-Mondragon JA, Riudavets-Puig R, Rauluseviciute I *et al.* JASPAR 2022: the 9th release of the open-access database of transcription factor binding profiles. *Nucleic Acids Res* 2022; **50**:D165-D173.
