# Supplementary material for: The developmental and evolutionary characteristics of transcription factor binding site clustered regions based on an explainable machine learning model
Source: Nucleic Acids Res. 2024 May 30;52(13):7610–26. doi: 10.1093/nar/gkae441 (PMC11260490; doi:10.1093/nar/gkae441)
Supplement: gkae441_Supplemental_Files [file gkae441_supplemental_files.zip › Supplementary_files_of_feature_statistics.tar.gz!/feature_statistics.chicken.HH11.feature0.html]

Catboost metrics graph
